# Antimicrobial Proteinoid Nanostructures via Thermal Condensation of L-Glutamic Acid and L-Tyrosine

**DOI:** 10.3390/nano15241846

**Published:** 2025-12-08

**Authors:** Marta Cadeddu, James R. G. Adams, Roberto La Ragione, Daniel K. Whelligan, Vlad Stolojan, Nadia Bernardi, Ioannis Smyrnias, Barbara Poddesu, Giulia Cugia, Davide De Forni, Luca Malfatti, Davide Carboni, Alessandra Pinna, Plinio Innocenzi

**Affiliations:** 1 Laboratory of Materials Science and Nanotechnology, CR-INSTM, Department of Engineering, University of Sassari, Via Vienna 2, 07100 Sassari, Italy; marta.cadeddu@dottorandi.unipg.it (M.C.); luca.malfatti@uniss.it (L.M.); 2 Discipline of Comparative Biomedical Sciences, School of Veterinary Medicine, Faculty of Health and Medical Sciences, University of Surrey, Guildford GU2 7AL, UK; j.r.adams@surrey.ac.uk (J.R.G.A.); r.laragione@surrey.ac.uk (R.L.R.); n.bernardi@surrey.ac.uk (N.B.); i.smyrnias@surrey.ac.uk (I.S.); 3 Discipline of Microbes, Infection and Immunity, School of Biosciences, Faculty of Health and Medical Sciences, University of Surrey, Guildford GU2 7YH, UK; 4 School of Chemistry and Chemical Engineering, University of Surrey, Guildford GU2 7YH, UK; d.whelligan@surrey.ac.uk; 5 Advanced Technology Institute (ATI), School of Computer Science and Electronic Engineering, University of Surrey, Guildford GU2 7YH, UK; v.stolojan@surrey.ac.uk; 6 ViroStatics S.r.l., Viale Umberto I, 46, 07100 Sassari, Italy; b.poddesu@virostatics.com (B.P.); g.cugia@virostatics.com (G.C.); d.deforni@virostatics.com (D.D.F.); 7 Department of Materials, Imperial College London, London SW7 2BX, UK

**Keywords:** proteinoids, antimicrobial peptides, L-glutamic acid, L-tyrosine, nanomaterials, *Staphylococcus*

## Abstract

The synthesis of biocidal peptide materials using simple, low-cost, solvent-free methods is a crucial challenge for developing new antimicrobial approaches. In this study, we produced proteinoid nanostructures through simple, inexpensive, and environmentally friendly thermal reactions between glutamic acid (Glu) and tyrosine (Tyr) in various molar ratios. Mechanistically, the thermal cyclization of glutamic acid into pyroglutamic acid (pGlu) facilitated the formation of short peptide chains containing pGlu as the *N-terminus* moiety and subsequent L-tyrosine or glutamic acid residues, which self-assembled into nanometric spheroidal structures that exhibit blue emission. Spectroscopic (FTIR, UV-Vis, photoluminescence) and mass (LC-MS) analyses confirmed the formation of mixed pGlu-/Tyr/Glu peptides. All products exhibit dose-dependent antimicrobial activity against Methicillin-Resistant *Staphylococcus aureus* (MRSA), with a minimum inhibitory concentration (MIC) of 25 mg mL^−1^ for the GluTyr 1:1 and 2:1 proteinoids. The outcomes observed following 24 h exposure of the HEK293 cell line to the materials indicate their suitability for integration into hybrid systems for antimicrobial surfaces. This work is the first to demonstrate a direct antibacterial activity of proteinoids obtained by thermal condensation, opening up the possibility of designing a new class of synthetic antimicrobial peptides.

## 1. Introduction

The growing global health emergency caused by antimicrobial resistance has driven research into new therapies for treating bacterial infections over the past two decades. Extensive and often inappropriate antibiotic use in both human and veterinary medicine has promoted the selection of multidrug-resistant strains. As a result, many traditional antibiotics are now ineffective, leading to increased illness and healthcare costs associated with infections that were once easily treated [1]. Among the pathogens of particular concern is Methicillin-Resistant *Staphylococcus aureus* (MRSA), which is resistant to conventional treatments and is a primary cause of nosocomial and community infections [2]. In response to this challenge, antimicrobial peptides (AMPs) have emerged as promising alternatives to traditional antibiotics [3]. These molecules are either naturally occurring, produced by bacteria, fungi, plants, and animals as components of their innate immune systems, or synthetically engineered in laboratory settings, where they are produced using chemical or recombinant DNA methods [4]. AMPs typically have an amphipathic structure and a net positive charge, which facilitates electrostatic interactions with bacterial membranes that are rich in anionic phospholipids [5]. This interaction can result in pore formation, membrane disruption, and cell death. AMPs also have the ability to interfere with intracellular processes, such as protein synthesis or DNA replication, and are generally active against a broad spectrum of microorganisms [6,7]. Despite their proven efficacy *in vitro*, AMPs have certain limitations that hinder their large-scale production and clinical application. The limitations include poor stability in physiological environments (especially in the presence of proteases), constraints in systemic transport due to their high susceptibility to enzymatic degradation, nonspecific cytotoxic effects, and high synthesis costs [8,9]. An interesting approach capable of overcoming some of these drawbacks can be achieved by synthesizing a new class of low-cost peptides, known as proteinoids, that can be designed as peptidomimetic nanostructures featuring AMP-like activity. Proteinoids represent an interesting class of synthetic peptide-based materials that can be obtained through thermal condensation reactions between α-amino acids. These reactions do not require catalysts or organic solvents. The technique, already recognized for its prebiotic potential, has recently regained interest in the biotechnology field due to its simplicity and sustainability [10]. The operating conditions—moderate temperatures (160–200 °C), short times (as low as 30 min), and absence of toxic reagents [11]—make this method easily scalable, cost-effective, safe, and compatible with biomedical applications [12,13]. The proteinoids obtained have a heterogeneous composition, which depends on the relative reactivity of the precursor amino acids. The presence of hydrophilic and hydrophobic portions, resulting from the side chains of the reacted amino acids, enables self-assembly in aqueous solution, and this feature is one of the most exploited for bio-applications [14,15]. A key element in the thermal synthesis of proteinoids is glutamic acid. Under heating, this amino acid goes through intramolecular cyclization to form pyroglutamic acid (pGlu) [16]. This conversion is essential since it triggers amidation reactions, thereby promoting the growth of peptide chains. In fact, melted pyroglutamic acid acts as a dispersing agent, promoting the homogenization of the fused solid mixture and enhancing molecular diffusion [17]. In our previous studies, we have demonstrated the effectiveness of pGlu in facilitating the polymerization of thermally unstable or non-reactive amino acids such as lysine or alanine, making it a fundamental component in the rational design of new proteinoids with non-aromatic fluorescent blue emission [18,19]. In this study, we focused particularly on the intramolecular cyclization of glutamic acid into pGlu and the thermal condensation reaction between L-glutamic acid (Glu) and L-tyrosine (Tyr) to produce nanostructured proteinoids that mimic the structural and functional features of AMPs. To reduce compositional complexity and enhance interpretability, the system was deliberately restricted to only two amino acid precursors. This minimalistic approach enables the generation of simplified proteinoid frameworks with reduced structural variability. Tyrosine was selected primarily for its aromatic character, which contributes to the optical and biological behavior of the resulting materials. L-tyrosine, however, has a high thermal decomposition temperature [20], and below ~280 °C, is relatively stable and does not polymerize on its own. To react at lower temperatures, it is necessary to have an initiator, such as L-glutamic acid, which also participates in the formation of the proteinoid structure. We have thus decided to apply the know-how previously acquired in developing fluorescent non-aromatic proteinoids to develop an easy and cost-effective procedure for synthesizing new proteinoids incorporating the L-tyrosine aromatic amino acids. The optical properties of the proteinoids obtained by L-glutamic acid and L-tyrosine were analyzed and compared to those of analogous proteinoids synthesized from non-aromatic amino acids such as lysine and alanine, which similarly lack the ability to thermally homopolymerize but feature aliphatic side chains [18,19]. Moreover, tyrosine contributes actively to the functionalization of the proteinoid system. Tyrosine-derived polymers are, in fact, a promising platform with successful application in the biomedical field, including biosensing, drug delivery, and tissue engineering. The phenol moiety participates in redox reactions, hydrogen bonding, and hydrophobic interactions, while also promoting nanostructure self-assembly via π–π stacking [21,22]. Tyrosine plays a functional role in enhancing the antibacterial activity of various molecular and nanostructured systems, largely due to the chemical versatility of its phenolic side chain. The phenol group can engage in hydrogen bonding and hydrophobic interactions, all of which are relevant to the direct contact with membrane phospholipids and to their disruption [23,24,25]. The thermal copolymerization of L-glutamic acid and L-tyrosine, therefore, enables the production of amphipathic proteinoids with inherent antibacterial properties, characterized by a controlled molecular architecture and well-defined optical properties. This simple synthetic strategy allows for the production of a new class of antimicrobial peptide-like materials, offering a minimalist but efficient platform for bioactive material design.

## 2. Materials and Methods

### 2.1. Chemicals

L-glutamic acid (C_5_H_9_NO_4_, Glu ≥ 99%) and L-tyrosine (C_9_H_11_NO_3_, Tyr ≥ 99%) powders were purchased from Sigma-Aldrich, St Louis, MO, USA. All chemicals were used as received without any further purification. Milli-Q water was used for purification and analysis.

### 2.2. Materials Synthesis

Three samples of proteinoids were synthesized by thermally treating a mixture of glutamic acid and tyrosine, changing their molar ratios but keeping the total weight at 4 g.

GluTyr 1:1: 1.79 g (0.012 mol) of glutamic acid and 2.21 g (0.012 mol) of tyrosine were mixed in a mortar. The resulting mixture was then placed in a 250 mL round-bottom flask and treated in an oil bath at 180 °C for 4 h under stirring at 250 rpm. The flask was set in the oil bath after the oil had reached the specified temperature. After cooling down at room temperature, 20 mL of Milli-Q water was added to the resulting dark-brown glassy mass, and the flask was sonicated for 20 min. The suspension was poured into a 50 mL Falcon tube, and another 10 mL of Milli-Q water was added to the flask to recover further material, pouring this into the Falcon tube, thus reaching 30 mL in total after sonicating again for 20 min. The suspension was filtered through cellulose acetate syringe filter (Whatman Puradisc 30, Sigma-Aldrich) with a pore size of 0.45 µm, obtaining a yellow-orange solution that was dialyzed against Milli-Q water (stirring at 350 rpm) using benzoylated membranes for dialysis tubing (avg. flat width 32 mm, 1.27 in., molecular weight cut-off = 2000 Da, Sigma-Aldrich) for 3 days; the dialysis water was replaced twice a day. Finally, the purified solution was freeze-dried with a Lio 5P, obtaining a yellowish powder.

GluTyr 2:1: 2.475 g (0.017 mol) of glutamic acid and 1.525 g (0.008 mol) of tyrosine were mixed in a mortar and then treated following the same procedure described for GluTyr 1:1.

GluTyr 4:1: 3.06 g (0.021 mol) of glutamic acid and 0.96 g (0.005 mol) of tyrosine were mixed in a mortar and then treated following the same procedure described for GluTyr 1:1.

### 2.3. Materials Characterization

UV–Vis absorption spectra were recorded using a Nicolet Evolution 300 UV–Vis spectrophotometer (Thermo Fisher Scientific, Madison, WI, USA) with a bandwidth of 1.5 nm.

Photoluminescence spectroscopy measurements were performed on a “NanoLog” Horiba Jobin Yvon spectrofluorometer (HORIBA Scientific, Kyoto, Japan). The 3D photoluminescence (PL) maps of aqueous solutions were recorded from 300 to 700 nm (slit 3 nm, integration time 0.1 s). The same spectrofluorometer and identical parameter settings were used in all the analyses.

Fourier-transform infrared (FTIR) analysis was carried out using an infrared Vertex 70 interferometer (Bruker Optics GmbH, Ettlingen, Germany). The FTIR absorption spectra were recorded in the 4000–400 cm^−1^ range with a 4 cm^−1^ resolution and 128 scans. The spectra were acquired using a potassium bromide pellet (1:100 weight ratio; KBr, ≥99.5%, Fluka, Sigma–Aldrich, Buchs, Switzerland). Spectra were analyzed using Opus 6.5 software with a rubberband function for baseline correction. The deconvolution of the FTIR bands in the range 1780–1570 cm^−1^ was performed through a Gaussian fit of the FTIR spectrum through OriginPro 2021 software, and the fit provided an associated R^2^ equal to 0.9997.

High-resolution LC-MS chromatograms and spectra were recorded using an Agilent 1260 Infinity II HPLC (Agilent Technologies, Santa Clara, CA, USA) coupled to an Agilent 6550 iFunnel QTOF mass spectrometer (Agilent Technologies, Santa Clara, CA, USA) using electrospray ionization (Jet Stream Technology, Agilent Technologies, Santa Clara, CA, USA) in negative mode. LC conditions used an Agilent Poroshell 120 EC-C18 column (Agilent Technologies, Santa Clara, CA, USA) (100 mm × 2.1 mm, 2.7 μm particle size), column temp. 30 °C, flow rate 0.30 mL min^−1^, mobile phase Solvent A: water, Solvent B: acetonitrile, gradient 0–1 min, 5% B; 1–9 min, 5–100% B; 9–10 min, 100% B; 10–11.5 min, 100–5% B; 11.5–12 min, 5% B. Agilent MassHunter Qualitative Analysis was used with the Molecular Feature Extraction (MFE) method to extract peaks representing compounds from the total ion count (TIC) chromatogram with their associated masses. Compounds with volumes (an Agilent property related to peak area but weighted by retention time and *m*/*z*) less than 100,000 were deleted. Compounds identified in the blank and the sample were compared, and matching compounds with *m*/*z* within 0.0005 Da and RT within 0.15 min were deleted from the sample compound list.

The same experimental conditions were used for pGlu quantification in all GluTyr products. All blanks, calibration standards, and samples were analyzed by LC-MS in triplicate. Agilent MassHunter Quantitative Analysis (for ToF) version 10.1.733.0 was used for the quantification of pyroglutamic acid with the following settings: retention time 0.886 min (left delta 0.4 min, right delta 0.6 min), *m*/*z* extracted 128.0371 ± 50 ppm.

NMR spectra were obtained on a Bruker 500 MHz spectrometer in DMSO-d6. ^1^H-NMR spectra were referenced to tetramethylsilane (TMS) at 0 ppm.

TEM/STEM samples were prepared by filtering a drop of sample solution through a holey-carbon 300 mesh Cu grid (Agar Scientific Ltd., Stansted, Essex, UK) and allowed to dry. The samples were imaged in the Thermo Scientific Talos 200Fi (Thermo Fisher Scientific, Waltham, MA, USA) in both transmission and scanning transmission modes at 200 keV. FIJI was used to obtain statistical particle size. To determine the nanoaggregate diameter, a line was drawn manually along the aggregates, and the length in pixels of this line was converted to nanometers using the scale bar. In total, 85 nanoaggregates within the GluTyr 1:1 sample were analyzed.

### 2.4. Bacterial Isolates and Growth Conditions

Methicillin-Resistant *Staphylococcus aureus* (MRSA) NCTC 12493 was stored in Pro-Lab Diagnostics Microbank (Fisher, Basingstoke, UK) tubes at −80 °C and then streaked onto LB Agar (Oxoid, Basingstoke, UK) and incubated at 37 °C aerobically for 18 h.

### 2.5. Bacterial Growth Curves

Briefly, 10 mL of Mueller–Hinton Broth 2 (MHB-2) (Oxoid, Basingstoke, UK) in a 50 mL Falcon tube was inoculated with a single bacterial colony using a sterile loop and incubated for 18 h, shaking at 200 RMP at 37 °C. The bacterial suspension was then adjusted with fresh MHB-2 until a turbidity equivalent to that of a 0.5 McFarland standard was reached, and 100 µL of this bacterial suspension was added to a 96-well plate containing 100 µL of proteinoids dissolved in MHB-2 at a fixed concentration of 10 mg mL^−1^. Plates were incubated aerobically for 18 h at 37 °C using a TECAN SPARK plate reader (Tecan Group Ltd., Männedorf, Switzerland), and readings were set every 30 min at OD_600_ nm. The compounds were tested in triplicate. Additionally, three wells were filled with 200 µL of bacterial suspension to act as growth control, and the other three wells were filled with 200 µL of media without bacterial inoculation to act as sterile control. The statistical analysis was performed by calculating the mean area under curve (AUC) for each curve and comparing all treatment groups to the growth control using one-way ANOVA followed by Dunnett’s post hoc test.

### 2.6. Minimum Inhibitory Concentration (MIC)

Minimum inhibitory concentrations (MICs) were determined using the broth microdilution method. Briefly, 10 mL of Mueller–Hinton Broth 2 (MHB-2) in a 50 mL Falcon tube was inoculated with a single bacterial colony using a sterile loop and incubated for 18 h, shaking at 200 RMP at 37 °C. The bacterial suspension was then adjusted with fresh MHB-2 until a turbidity equivalent to that of a 0.5 McFarland standard was reached, and 100 µL of this bacterial suspension was added to a 96-well plate containing 100 µL of test compounds, which had been diluted two-fold in MHB-2 to generate a range of concentrations from 25 to 0.05 mg mL^−1^. Additionally, 100 µL of bacterial suspension was added to a well containing 100 µL of media alone to act as a growth control. A well with 200 µL of media without bacterial inoculation acted as a sterile control. The experiment was conducted in triplicate. Plates were incubated aerobically for 18 h at 37 °C. After incubation, MIC was determined through visual observation of no turbidity within the media in the well and confirmed using a TECAN SPARK plate reader (Tecan Group Ltd., Männedorf, Switzerland) at OD6_00_ nm, where inhibition was characterized as OD_600_ nm < 50% of the triplicate positive control. Statistical analysis was performed using a two-way ANOVA followed by Dunnett’s post hoc test, comparing each concentration to the growth control (0.00 mg mL^−1^).

### 2.7. Cell Line

The cytotoxicity of GluTyr proteinoids was studied in HEK293 cells (human embryonic kidney, ATCC, CRL-1573). The cell line was routinely maintained in a Dulbecco Modified Eagle Medium (DMEM) supplemented with 1% glutamine, 1% penicillin/streptomycin, and 10% fetal bovine serum (FBS), and used to assess the cytotoxicity of the compounds.

### 2.8. Cytotoxicity

The cytotoxicity was determined by a standard MTS (3-(4,5-dimethylthiazol-2-yl)-5-(3-carboxymethoxyphenyl)-2-(4-sulfophenyl)-2H-tetrazolium) assay (CellTiter 96^®^ Aqueous One Solution Reagent, Promega Corporation, Madison, WI, USA). HEK293 cells were seeded at a density of 20.000 cells/well into a 96-well plate in DMEM supplemented with 1% glutamine, 1% penicillin/streptomycin, and 10% fetal bovine serum (100 µL each well) and incubated at 37 °C and 5% CO_2_. The day after the seeding, the media were replaced with fresh DMEM supplemented with 1% glutamine as a control (100 µL each well) and with different concentrations of the GluTyr proteinoids (from 25 to 0.2 mg mL^−1^ with 2-fold dilution) dissolved in fresh DMEM supplemented with 1% glutamine (100 µL each well). Cells were exposed to the proteinoids for 24 h at 37 °C and 5% CO_2_, and then cell viability was measured with the MTS assay. In this assay, 20 µL of MTS reagent was added to each well, and the plates were incubated for 2 h at 37 °C and 5% CO_2_. THE TECAN SPARK plate reader (Tecan Group Ltd., Männedorf, Switzerland) was used to measure absorbance at 490 nm. The experiment was performed in technical duplicate for each compound tested. Raw data were analyzed using GraphPad Prism 9.0, normalizing to the untreated control. Dose–response curves were fitted using non-linear regression with variable slope (four-parameter model), following log transformation of the X-axis, to calculate CC_50_ values. R^2^ values were around 0.95 for GluTyr 1:1 and 0.97 for GluTyr 4:1. To compare the potency of the two treatments (GluTyr 1:1 vs. GluTyr 4:1), an extra sum-of-squares F-test was used to determine if the Log(CC_50_) parameter was significantly different between the two datasets.

## 3. Results and Discussion

Pursuing the study of low-temperature and solvent-free copolymerization of amino acids mediated by pyroglutamic acid, we assessed the feasibility of this synthetic approach by performing reactions between glutamic acid and tyrosine at 180 °C at various molar ratios.

**Scheme 1:**
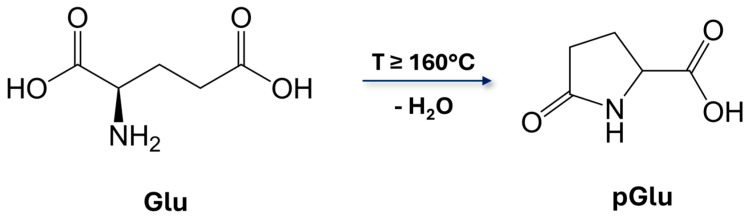
Intramolecular dehydrocondensation reaction of L-glutamic acid (Glu) into pyroglutamic acid (pGlu).

In our earlier studies [19,26], we have extensively investigated how L-glutamic acid (Glu) and its heat-formed cyclic derivative, pyroglutamic acid (pGlu), work as dispersing agents and initiators for amidation reactions without solvents at temperatures as low as 160 °C. Upon heating, Glu undergoes melting and intramolecular cyclization to form γ-lactam pGlu (Scheme 1).

This compound acts as a reactive intermediate, facilitating nucleophilic attack by primary amines, thereby initiating the step-growth polymerization pathway. Besides its chemical reactivity, converting Glu into melted pGlu favors a better mixing of the precursors. This makes amidation reactions easier and allows amino acids like L-tyrosine (Tyr), which do not homopolymerize at low temperatures, to attach. As a result, the polymer grows more uniformly, and the proteinoid synthesis is more reproducible. These properties make Glu and pGlu not only chemically efficient but also strategically advantageous components in the design of such peptido-mimetic materials.

**Figure 1:**
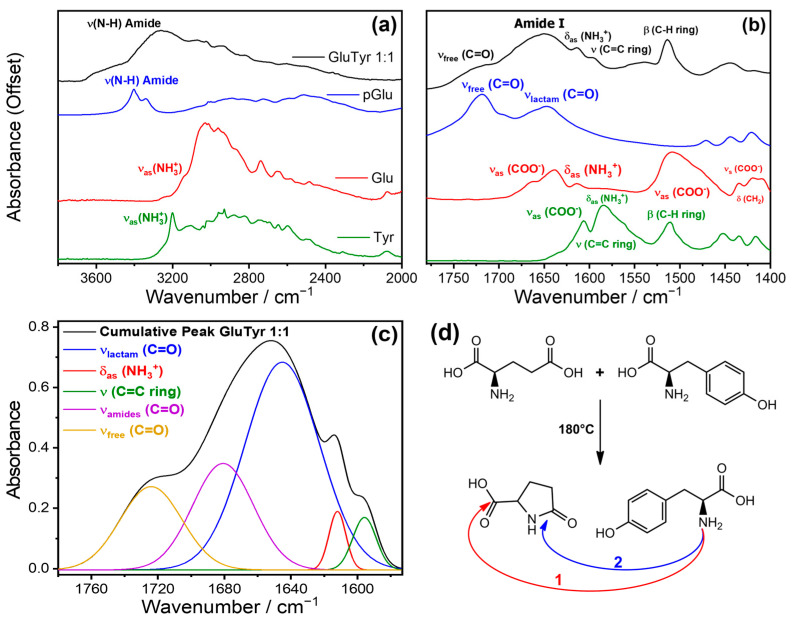
FTIR absorption spectra of L-tyrosine (Tyr) (green line), L-glutamic acid (Glu) (red line), pyroglutamic acid (pGlu) (blue line), and GluTyr 1:1 (black line) in the following range: (**a**) 3800–2000 cm^−1^ and (**b**) 1775–1400 cm^−1^. (**c**) Gaussian deconvolution with five components of the Amide I region in the range 1780–1570 cm^−1^. (**d**) Scheme of the plausible reaction pathway: Glu-to-pGlu conversion and possible nucleophilic attack at two sites, promoting linear (1) or ring-opening (ROP) polymerization (2).

To assess the thermally induced formation of peptide chains that have incorporated tyrosine units, FTIR analysis was performed (Figure 1).

Figure 1a (3800–2000 cm^−1^) and Figure 1b (1775–1400 cm^−1^) show the FTIR spectra of the unreacted precursors (Tyr (green line), Glu (red line)), the intermediate pGlu (blue line) and the compound resulting from the thermal reaction between Tyr and Glu (GluTyr 1:1, black line) at 180 °C, followed by filtration, dialysis, and freeze-drying. The GluTyr spectrum exhibits a primary component characterized by a broad absorption band centered at 3271 cm^−1^ that is consistent with amide N-H stretching ν(N-H) of peptide bonds. The wide shoulder near ~3540 cm^−1^ is assigned to weakly H-bonded OH in surface water/tyrosine. These bands are shifted and broadened with respect to the ones related to the asymmetrical stretching modes ν_as_ (NH_3_^+^) of the unreacted amino acids [27] (Glu and Tyr) and the ν(N-H) of pGlu amide. pGlu shows two overlapped bands, at 3337 cm^−1^ and at 3402 cm^−1^, related to the ν(N-H) stretching due to the formation of the internal amide (lactam) [28] (Figure 1a). The broadness of the GluTyr bands in the range 3500–3200 cm^−1^ is attributable to the heterogeneous distribution of H-bonds in a proteinoid and the possible presence of residual adsorbed water. This characteristic could also suggest the presence of different isomers resulting from the amidation between Glu and Tyr.

In the case of GluTyr 1:1, the main evidence for amide bonds is the presence of a very broad typical primary amide stretching ν(-C=O) centered at 1649 cm^−1^ (Amide I) [29,30,31]. The aforementioned band is maintained in all three final products whose profiles appear nearly perfectly superimposable (Figure S1a, Supporting Information), indicating only minimal structural variations between the samples—differences that are challenging to detect using this spectroscopic method. The intermediate pGlu shows two different bands, the first related to the ν(-C=O) stretching of the free carboxyl group at 1718 cm^−1^ and the other related to the ν(-CONH-) stretching due to the formation of an internal amide (lactam) at 1647 cm^−1^ [28] (Figure 1b). To resolve the broad Amide I band of GluTyr 1:1, a Gaussian deconvolution with five components was performed on the spectral range spanning from 1780 to 1570 cm^−1^ (Figure 1c). This allows us to differentiate, in the amide range, the spectral contribution of the peptide bonds formed after thermal treatment from that derived from pGlu, which may have been integrated in the final proteinoids. The analysis of the deconvolution highlights the presence of some residual unreacted carboxyl groups (ν_free_(-C=O)) at 1724 cm^−1^, the pGlu lactam ((ν_lactam_(-C=O)) at 1645 cm^−1^, the bending of free ammonium groups (δ_as_ (NH_3_^+^)) at 1612 cm^−1^ and the aromatic ring stretching ν(-C=C ring) at 1595 cm^−1^. A new amide band is centered at 1680 cm^−1^, which confirms new amide bonds formed after thermal condensation between Glu and Tyr. Additionally, the analysis shows the pGlu lactam stretching (ν_lactam_(-C=O)) at 1645 cm^−1^. The comparison with the tyrosine spectrum reveals good correspondence for the vibrational modes of the NH_3_^+^ bending (δ_as_(NH_3_^+^)) and the aromatic ring stretching (ν(C=C)) of tyrosine that are shifted, respectively, from 1606 and 1585 cm^−1^ to 1614 cm^−1^ and 1596 cm^−1^ of GluTyr products. Moreover, a good correspondence can be highlighted for the band at 1510 cm^−1^, ascribed to the C-H ring bending (β(C-H ring)) of tyrosine [31,32,33,34,35], which slightly shifts to 1514 cm^−1^ in GluTyr. On the other hand, the range from 1100 cm^−1^ to 1300 cm^−1^ of the GluTyr spectra seems to be very similar to that of pGlu, involving the bands at around 1230 cm^−1^, which can be related to the C-O stretching overlapping the Amide III band, and those around 1107 cm^−1^ associated with the lactam ring stretching [28] (Figure S1b, Supporting Information). These observations evidence the formation of peptide chains that undoubtedly incorporate both pGlu and tyrosine moieties. This finding is also confirmed by a deep analysis of the reaction mixture performed by LC-MS (*vide infra*).

**Figure 2:**
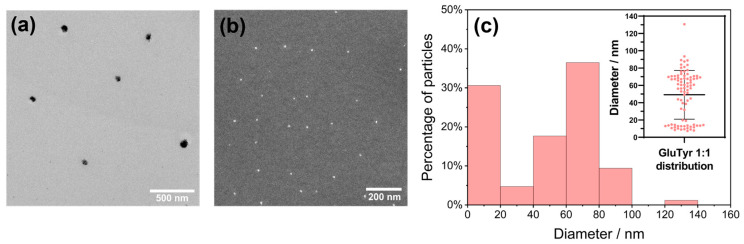
STEM images of GluTyr 1:1 in bright field with a scale bar of 500 nm (**a**) and in dark field with a scale bar of 200 nm (**b**). Histogram of the particle size distribution of GluTyr 1:1 obtained from dimensional analysis with FIJI (**c**). Bars represent the percentage of particles relative to the total count within each size interval. The inset graph shows the scatter plot of the dimensional distribution of GluTyr 1:1. Data points represent every singular nanoaggregate of the 85 nanoaggregates whose diameter was estimated with FIJI. Mean (49.12 nm) and standard deviation (29.12 nm) are indicated, respectively, with a bold horizontal line and error bar.

The formation of particle-like nanostructures by these proteinoids was studied by scanning transmission electron microscopy (STEM). Previous studies have demonstrated that proteinoids can spontaneously self-assemble in water into spheroidal structures, typically ranging up to a few micrometers in diameter [14,36]. This feature is attributed to their chemical composition, since they contain both hydrophobic and hydrophilic regions depending on the sidechain of the amino acid precursors. The amphiphilic nature of proteinoids, resembling that of phospholipids in biological membranes, facilitates their aggregation into capsule-like structures [15,37]. Figure 2 shows the STEM images in bright (Figure 2a) and high-angle annular dark (Figure 2b) field of the GluTyr 1:1 product.

The sample morphology appears as spheroidal nanoaggregates, while the size distribution (Figure 2c) shows wide variability. Diameters range from approximately 10 to 130 nm. The histogram shows a multimodal distribution, characterized by a first population of smaller particles (<20 nm) and a second prevalent population between 60 and 80 nm in diameter. These results are very similar to those previously observed for proteinoids obtained through solvent-free thermal treatment of L-glutamic acid and L-lysine [2]. In the case of GluTyr 1:1, the hydrophobic regions related to the phenol ring of tyrosine are likely to be arranged within a spheroidal architecture through π–π interactions that minimize the interaction with the polar solvent, while the hydrophilic groups (-NH_2_, -COOH) are outwardly exposed. In this scenario, pyroglutamic acid may serve a dual role: it stabilizes the interface with polar solvent through hydrogen bonds [38] involving its carbonyl group, and at the same time, it introduces greater rigidity into the peptide chains due to the lactam ring that enhances aggregation phenomena. This is analogous to the role of the *N-terminus* proline or hydroxyproline 5-membered ring in tripeptides consisting also of two phenylalanine residues (an aromatic amino acid) studied by Bera et al., which have shown good aggregation properties involving head-to-tail hydrogen bonding [39]. Furthermore, cyclization results in the loss of the positive charge on the free amino group. This process increases the system’s hydrophobicity and promotes tighter chain packing [40].

A high-resolution LC-MS analysis was performed to elucidate the composition of the materials. This analysis revealed that the peptide component profiles of the products differed qualitatively, with these differences attributed to changes in reaction kinetics resulting from different molar ratios of precursor compounds.

**Figure 3:**
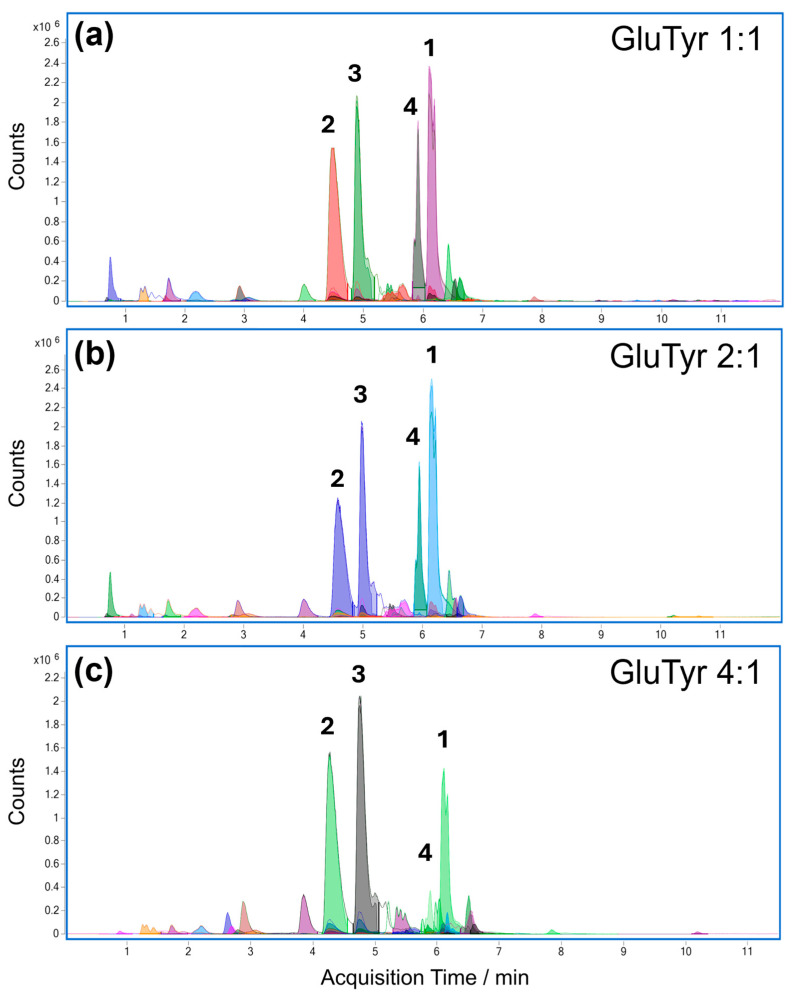
Extracted chromatograms (using Agilent MassHunter Molecular Feature Extraction) of compounds formed from Glu and Tyr reaction mixture in (**a**) 1:1, (**b**) 2:1, (**c**) 4:1 molar ratios, with the 4 most abundant compounds labeled in order of highest abundance.

Dedicated software was used to extract molecular features from the LC-MS data of the GluTyr 1:1 sample (see Table S1, Supporting Information). This analysis revealed the presence of several compounds (listed in Table S2, Supporting Information), although only four of these formed the majority, as shown in the chromatogram in Figure 3a.

**Scheme 2:**
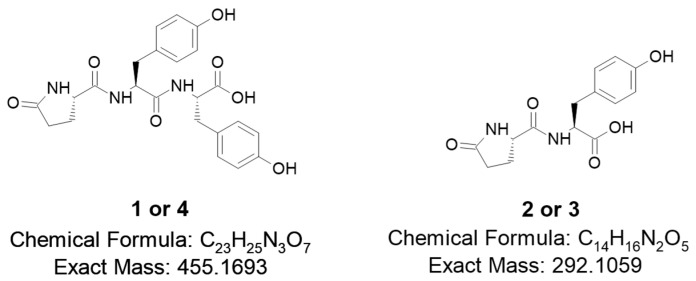
Possible structures corresponding to the 4 most abundant peaks identified by LC-MS. Multiple peaks with the same exact mass are most likely due to diastereoisomers.

The accurate masses of the compounds producing peaks 1 and 4 correspond to one residue of pGlu and two residues of Tyr, while those for peaks 2 and 3 correspond to one residue of pGlu and one of Tyr. Based on accurate mass measurements, the formation of diastereoisomers via epimerization of chiral centers under the reaction conditions is the likely explanation for peaks with the same exact mass but different retention times. Alternatively, the occurrence of isomeric species resulting from esterification of the phenolic group by pGlu is plausible, though the NMR (described below) indicates that this does not seem to be the case. The plausible structures related to the 4 most abundant compounds are described in Scheme 2.

Moreover, the LC-MS data allowed us to also evaluate the molecular weight distribution of our proteinoid mixtures (see Figure S2, Supporting Information), indicating that, for the GluTyr 1:1 and 2:1 samples, roughly 40% of the products are represented by the sum of diastereomers 2 and 3, and more than 35% are provided by the sum of diastereomers 1 and 4. In the case of the GluTyr 4:1 sample, the large excess of pGlu instead favored the formation of the dipeptide (>62%) while limiting the presence of the tripeptide to about 18%.

The ratio of the produced compounds and isomers may be affected by the molar ratio of the starting amino acids: while the 2:1 mixture gave a compound profile nearly identical to that of the 1:1 mixture (Figure 3b), the 4:1 mixture showed a significantly reduced peak for compound 4 (Figure 3c), which is one that does require more Tyr units than pGlu. This analysis further confirms the key role of glutamic acid in facilitating the polymerization of amino acids that, on their own, would not be able to form homopolymer chains. Its derivative, pyroglutamic acid, triggers the amidation reaction by acting both as a dispersing agent and a root for the subsequent attachment of additional amino acid units. In this case, the oligomeric chains formed are relatively short, the majority containing no more than three consecutive tyrosine residues linked by peptide bonds.

**Figure 4:**
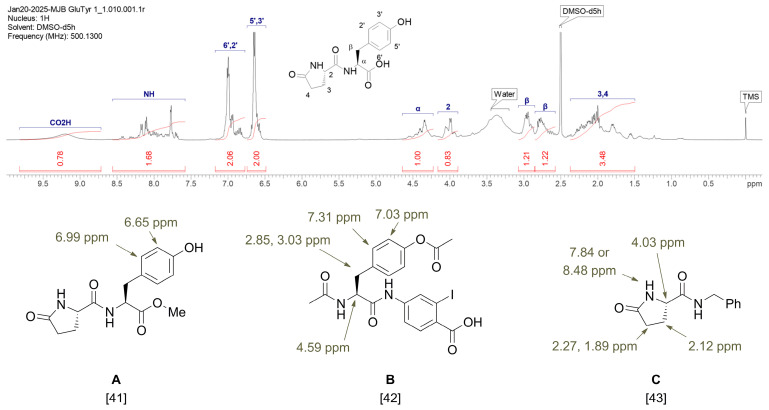
Top: ^1^H NMR spectrum of compound mixture resulting from reaction of Glu and Tyr (1:1). Bottom: published compounds with structures related to those proposed in the mixture and NMR analysis performed in DMSO-d6 [41,42,43].

^1^H NMR analysis of the peptide mixtures was also performed (in Figure 4, the top image shows the spectrum for the mixture derived from a 1:1 Glu:Tyr ratio) and indicated a complex mixture of overlapping multiplets.

Nevertheless, by comparison with the NMR spectrum of pGlu, D_2_O-shake and COSY NMR experiments (see Figures S3–S9, Supporting Information), and NMR data of literature compounds A–C [41,42,43] (Figure 4, bottom), assignments of the peaks could be performed and are given in the figure (see Supporting Information for a full explanation). Importantly, comparing compounds A and B, the acylation of tyrosine is shown to lead to deshielding of the *ortho-* and *meta*-protons by >0.3 ppm. These peaks of the peptide mixtures are found at 7.00 and 6.64 ppm, which correspond almost exactly to non-acylated Tyr. Thus, it is most likely that the major components of the peptide mixtures contain only amide bonds to pGlu and not ester bonds through tyrosine’s phenol. This agrees with the IR data (Figure 1b), where the deconvoluted amide peak at 1680 cm^−1^ is at a much lower wavenumber than that of an aryl ester. Of further interest is the ratio of the integrals for the pGlu -CH and the tyrosine 3′,5′-Hs, suggesting that the predominant components of the 1:1 reaction product mixture are dipeptide pGlu-Tyr isomers rather than the 53:47 mixture of dipeptide and pGlu-Tyr-Tyr tripeptide suggested by LC-MS. However, this might be explained by the increased ionizability of the tripeptide in the mass spectrometer. The 2:1 product mixture shows very similar integral ratios (see Figure S6, Supporting Information), but those for the 4:1 Glu:Tyr reaction product mixture (see Figure S8, Supporting Information) have a ratio of integrals, suggesting a lower overall presence of Tyr in the peptides, which agrees with the LC-MS data.

Following structural characterization, the optical properties of GluTyr proteinoids were investigated. In our previous studies, we demonstrated the origin of photoluminescence of thermally obtained proteinoid made by non-aromatic amino acids [18,19]. In this work, we address the optical features of such materials by embedding an aromatic amino acid that is itself able to absorb and emit in the UV-Vis region due to the presence of delocalized π electrons [44].

**Figure 5:**
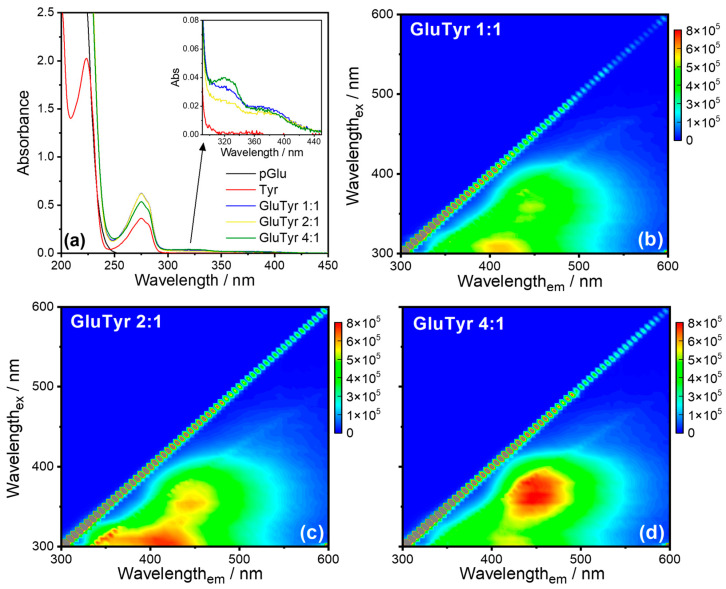
(**a**) UV–visible absorption spectra of 3 mg mL^−1^ of pGlu, 0,05 mg mL^−1^ of L-Tyr, and 0.125 mg mL^−1^ of GluTyr products’ aqueous solutions. The concentrations were selected not to saturate the UV absorption, keeping the maximum absorption value under 3. At the same time, the concentration must not be too low in order to enlighten the absorption peaks. The inset graph shows details of the absorption spectra in the 300–450 nm range; 3D-PL maps [excitation (y)–emission (x)–intensity (z)] in the 300–600 nm range of (**b**) GluTyr 1:1 0.5 mg mL^−1^, (**c**) GluTyr 2:1 0.5 mg mL^−1^, and (**d**) GluTyr 4:1 0.5 mg mL^−1^ aqueous solution.

Figure 5a shows the absorption spectra of the GluTyr products, along with tyrosine and pyroglutamic acid. The three absorption bands observed for tyrosine, located at approximately 200, 223, and 276 nm, are due to π to π* electronic transitions. Specifically, the bands at 223 and 276 nm correspond to the *S*_0_ to *S*_1_ and *S*_0_ to *S*_2_ state transitions, respectively, while the band near 200 nm is attributed to the *S*_0_ to *S*_3_ transitions [45]. In comparison, pGlu displays a main band at 202 nm (see Figure S10, Supporting Information), attributed to π–π* transitions associated with the carbonyl of the lactam ring and the free carboxylic group. All GluTyr proteinoids show a peak around 205 nm originating from the pyroglutamic acid component, as well as absorption bands at 223 nm (Figure S10, Supporting Information) and 276 nm characteristic of tyrosine. This indicates the presence of both tyrosine and pGlu units within the peptide chains, as initially suggested by FTIR characterization and later confirmed by LC-MS and ^1^H-NMR analysis. Additionally, the proteinoids exhibit a weak absorption at 320 nm, extending as a tail up to 440 nm. This feature is attributed to n-π* transitions characteristic of amide groups and, together with the close-packed arrangement of peptide chains, explains the observed blue emission [18,46].

The 3D fluorescence maps (x—emission; y—excitation; z—false color intensity scale) were acquired from 300 to 600 nm. Within this range, both pGlu and tyrosine (see Figure S11a,b, Supporting Information) do not show any significant emission.

On the other hand, GluTyr proteinoids exhibit blue emission with two distinct centers (Figure 5b–d): one at 415 nm (λ_ex_ = 315 nm) and another at 450 nm (λ_ex_ = 355 nm). Both emissions can be correlated to the n-π* transition observed in the UV spectra and are directly ascribed to the peptide bonds. The visible-range photoluminescence arises from both charge transfer and recombination processes involving the peptide backbone, which contains amide groups, and from short hydrogen bonds between closely packed peptide chains [47,48,49,50]. The same phenomena have been identified as accountable for the blue emission in lysine-based materials obtained with solvent-free thermal synthesis [51,52]. Notably, the second emission center is significantly more intense for the sample GluTyr 4:1, supporting the existence of structural differences between GluTyr samples as unveiled by LC-MS analysis.

Amphipathic molecules such as antimicrobial peptides use both hydrophilic and hydrophobic regions to target bacterial membranes and interact with their lipid bilayers. By doing so, they destabilize and disrupt the membrane structure, causing cell lysis and bacterial death [7]. Given their nanostructural aggregation and considering the molecular characteristics of the GluTyr products—particularly the amphipathic properties conferred by the presence of pyroglutamic acid and tyrosine—we considered GluTyr short-peptide nanoaggregates as promising antimicrobial candidates.

**Figure 6:**
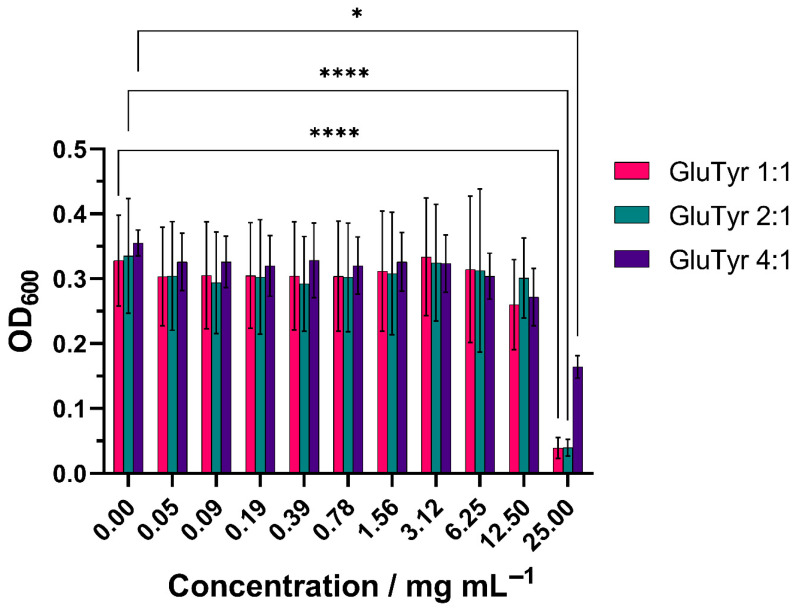
Minimum inhibitory concentration of GluTyr 1:1, GluTyr 2:1, and GluTyr 4:1 against MRSA determined by the broth microdilution method. Data are presented as the mean OD_600_ ± standard deviation (SD) of independent replicates. OD_600_ was measured after 18 h of incubation. Sterile control values referring to the media without any bacterial inoculation or proteinoids were subtracted as background. Statistical analysis was performed using a two-way ANOVA followed by Dunnett’s post hoc test, comparing each concentration to the growth control (0.00 mg mL^−1^). Asterisks indicate a statistically significant difference: * *p* < 0.05, **** *p* < 0.0001. The experiment was performed with a biological replicate of 2, and in 3 technical replicates to ensure data integrity and reproducibility.

Methicillin-Resistant *Staphylococcus aureus* (MRSA), a Gram-positive bacterium, was selected as the biological target since it has emerged as one of the leading causes of bacterial infections in both healthcare settings and community environments [2]. This strain of *S. aureus* has developed resistance to commonly used antibiotics, particularly beta-lactams like methicillin, making infections more difficult to treat and contributing to increased rates of illness and death [53]. We therefore exposed MRSA to GluTyr 1:1, 2:1, 4:1 proteinoids using the broth microdilution method [54] at a range of concentrations from 25 mg mL^−1^ to 0.05 mg mL^−1^, in order to assess their antimicrobial activity and determine a minimum inhibitory concentration (MIC). A growth control, consisting of media with bacterial inoculation without any proteinoid (0.00 mg mL^−1^) and a sterile control, consisting of media without any bacterial inoculation or proteinoids, were used as references. The sterile control values were subtracted as background. The measured optical density at 600 nm (OD_600_) is directly correlated to bacterial cell density. As shown in Figure 6, both GluTyr 1:1 and GluTyr 2:1 effectively inhibited the growth of MRSA at a concentration of 25 mg mL^−1^. GluTyr 4:1 also exhibited an inhibitory effect at this concentration, although it was less pronounced. These effective concentrations against MRSA are significantly higher than those of reference antibiotics, as well as other natural and synthetic antimicrobial peptides, which typically act at concentrations of a few hundred µg mL^−1^ to as low as <1 µg mL^−1^ [55,56,57]. However, a direct comparison is challenging and potentially misleading due to the nature of proteinoids, which are formed by a mixture of peptide chains of different lengths and isomers. Unlike pure antibiotics or antiseptics such as polyhexamethylene biguanide (PHMB), with defined molecular structure and targets [57], GluTyr proteinoids act as a polydisperse mixture of self-assembling nanostructures. These findings are nonetheless significant as they provide the first direct evidence of antibacterial activity from proteinoids produced by solvent-free thermal condensation.

**Figure 7:**
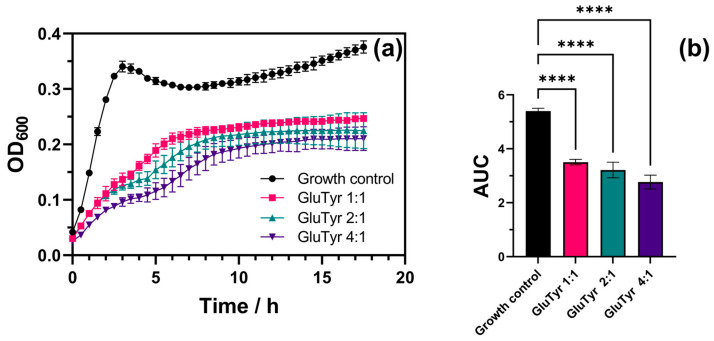
(**a**) MRSA growth curve exposed to GluTyr 1:1 (pink line), GluTyr 2:1 (green line), and GluTyr 4:1 (violet line) at 10 mg mL^−1^. Growth control (black line) refers to the media with bacterial inoculation without any proteinoid. Data are presented as the mean OD_600_ in function of time ± standard deviation (SD) of independent replicates. Sterile control values, referring to the media without any inoculation or proteinoids, were subtracted as background. The experiment was performed with a biological replicate of 2, and in 3 technical replicates to ensure data integrity and reproducibility. (**b**) The bar chart displays the mean area under the curve (AUC) calculated from the individual growth curves shown in (**a**). Columns represent the mean AUC ± standard deviation (SD). Statistical analysis was performed using a one-way ANOVA followed by Dunnett’s post hoc test, comparing all treatment groups to the growth control. Asterisks indicate levels of statistical significance: **** *p* < 0.0001.

We also studied how GluTyr products affect the growth kinetics of the selected MRSA strain. In this experiment, MRSA was exposed to a fixed concentration of 10 mg mL^−1^ of GluTyr 1:1, 2:1, and 4:1 and incubated for 18 h. Although this concentration was found to be sub-inhibitory in Figure 6, we hypothesized that it was still capable of affecting the bacterial growth kinetics. As shown in Figure 7, all three GluTyr proteinoids slowed bacterial proliferation, with the most pronounced effect observed during the exponential growth phase (1–4 h).

Pyroglutamic acid (2-pyrrolidone-5-carboxylic acid) has been widely studied for its antibacterial efficacy. This compound is produced by lactic acid bacteria, such as *Lactobacillus* and *Pediococcus*, as a metabolite with antimicrobial properties, and it has demonstrated inhibitory effects against several bacteria responsible for food spoilage. However, its mechanism of action is still unclear and can involve both the pH influence (behaving as an organic acid) and the interaction with bacterial membranes [58,59,60]. Its high efficacy and safety in aqueous formulations in combination with copper salts have led to the hypothesis of topical antibacterial applications against *Staphylococcus aureus* [61,62]. To determine whether any free pGlu remaining after dialysis could affect the antimicrobial results, we performed quantification of free pGlu in the samples using high-resolution LC-MS. The related calibration curve is reported in Figure S12, Supporting Information. The calculated concentrations of pGlu are reported in Table S3, Supporting Information.

Table 1 shows the calculated percentages of free pGlu with respect to GluTyr proteinoids (*w*/*w* %). Given the MIC of pGlu (6.5 mg mL^−1^, Figure S13, Supporting Information) compared to the higher MIC of GluTyr proteinoids (25 mg mL^−1^), the calculated percentages clearly demonstrated that only a negligible amount of free pGlu remains in the GluTyr samples after purification by dialysis. This provides strong evidence that the observed antibacterial activity arises exclusively from the peptide-based nanoaggregates and not from residual free pGlu. For example, in the case of GluTyr 1:1 at 25 mg mL^−1^, the concentration of free pGlu is only 0.13 mg mL^−1^, which is well below its MIC.

Many natural antimicrobial peptides work by interacting with bacterial cell membranes, leading to their permeabilization or destabilization [6]. We hypothesized that GluTyr proteinoids, due to the presence of hydrophobic residues and amphipathic features, may act similarly by promoting interaction with bacterial surfaces and membrane insertion, ultimately compromising cell integrity [7]. When proteinoids assemble into nanostructures, rather than existing as individual molecules, they can amplify their amphipathic properties by presenting a multivalent surface for membrane contact. This may increase the active material’s surface that is available to interact with the bacterial membrane, improving the antimicrobial efficacy. The nanostructure arrangement could also offer additional advantages such as increased resistance to enzymatic degradation, improved stability, and enhanced antimicrobial efficacy [8]. From a chemical perspective, tyrosine—with its phenolic side group—can integrate into the interfacial regions of membrane proteins, allowing for interaction with polar–apolar interfaces of membranes [25,63,64]. Pyroglutamic acid (pGlu) moiety enhances the hydrophobicity of the system due to its lactam ring, likely providing a significant contribution to the amphipathic properties of the proteinoid [39]. Furthermore, pGlu is present at the *N-terminus* of the synthetized peptides; this is a recurring post-translational modification in proteins and has been associated with increased stability against proteolytic degradation and, in some cases, with additional bioactive properties [65,66].

**Figure 8:**
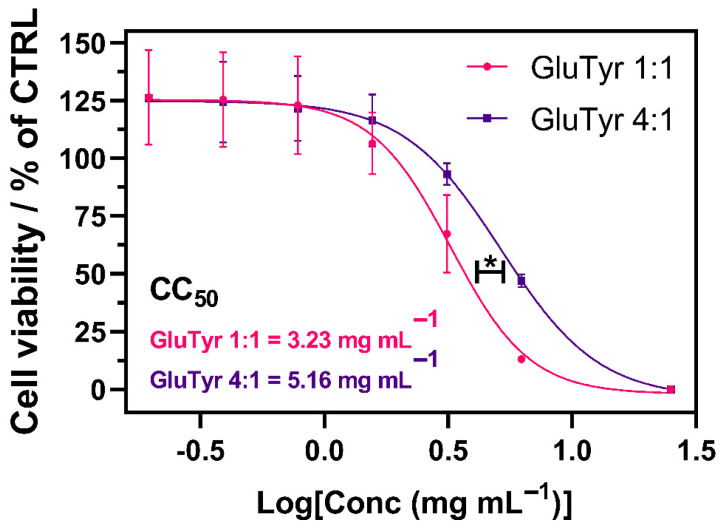
Cytotoxicity of GluTyr 1:1 and GluTyr 4:1 from MTS cell viability assay as a function of concentration. Cells were incubated with MTS reagent for 2 h before readings. The experiment was performed in 2 technical replicates to ensure data integrity and reproducibility. Cell viability is normalized to the untreated control. Dose–response curve was fitted by non-linear regression with variable slope (four-parameter model), after log transformation of the X-axis. The asterisk (*) indicates a statistically significant difference between the Log(CC_50_) values of the two formulations (*p* < 0.05, extra sum-of-squares F-test).

The biocompatibility of GluTyr proteinoids was evaluated through a typical MTS assay on the HEK293 cell line after 24 h of exposure. We selected GluTyr 1:1 and GluTyr 4:1 for evaluation, given the similarity between GluTyr 1:1 and GluTyr 2:1 samples and the differences revealed by GluTyr 4:1 in terms of peptide composition, optical properties, and MIC. As shown in Figure 8, both products exhibit a dose-dependent and pH-independent cytotoxicity (see Figure S14, Supporting Information), with calculated CC_50_ values of 3.23 mg mL^−1^ and 5.16 mg mL^−1^ for GluTyr 1:1 and GluTyr 4:1, respectively. A significant reduction in cell viability was observed at concentrations above 10 mg mL^−1^, which overlaps with the concentration required for antibacterial efficacy. In fact, the dose–response curve indicates a CC_50_ of 3.23 mg mL^−1^ for GluTyr 1:1, which is markedly lower than the MIC of 25 mg mL^−1^. Consequently, cell viability is negligible at the concentration required to completely inhibit bacterial growth. This cytotoxicity is comparable to that observed with broad-spectrum antimicrobial peptides such as melittin, the main component of bee venom [67], that similarly display reduced selectivity between bacterial and eukaryotic membranes. This result confirms that, while the material has antimicrobial potential, its narrow therapeutic window in the free form precludes systemic administration. Although the cytotoxicity data indicate a limitation for the direct use of GluTyr proteinoids in cellular applications, their high antibacterial efficacy, particularly against resistant strains like MRSA, remains a notable advantage. These findings suggest that GluTyr proteinoids could be applied in non-cellular settings, where their antimicrobial properties can be utilized without compromising biological safety. For example, embedding the proteinoids in hybrid materials or surfaces that are designed to confine the active materials, minimize systemic exposure, and maintain antibacterial effectiveness.

## 4. Conclusions

In this work, we demonstrated the feasibility of a solventless thermal reaction between glutamic acid and tyrosine to form a new class of antimicrobial peptides with a nanostructured architecture. The obtained proteinoids consist of a mixture of short peptides, with the major components composed of one pyroglutamic acid unit and up to three tyrosine moieties. The current results, together with previous studies, confirm the key role of pyroglutamic acid as the precursor monomer in enabling the homo-oligomerization of amino acids, which tend to decompose at high temperatures instead of polymerizing. Such peptide-based materials exhibit a nanometric spheroidal morphology, favored by the material’s hydrophilic and hydrophobic moieties, which are characteristic of these proteinoids. The composition of the GluTyr products was found to be affected by the molar ratio between the starting amino acids, as shown by LC-MS, and these diversities were reflected in the optical properties of the materials. Indeed, the products exhibit a double emission in the blue region of the visible spectrum, related to charge transfer and charge recombination phenomena, as well as short hydrogen bonds involving the amide groups of the packed peptide chains within the nanoaggregate. For the first time, a direct antibacterial activity was observed for proteinoids obtained by solventless thermal condensation. At a concentration of 10 mg mL^−1^, all GluTyr samples with varying Glu and Tyr molar ratios slowed MRSA growth. Notably, GluTyr 1:1 and 2:1 completely inhibited MRSA growth at 25 mg mL^−1^. The observed antibacterial activity is likely to be attributed to the amphiphilic nature of the peptides, which promotes their interaction with and disruption of bacterial membranes. While cytotoxicity was observed in HEK293 cells at the concentrations required for antibacterial activity, this limitation could be addressed by immobilizing, encapsulating, or incorporating the proteinoids into coatings, implant surfaces, and controlled release systems, allowing for safe biomedical applications. Various strategies for integration into hybrid or composite systems can be adopted to mitigate toxicity while maintaining effective antibacterial activity. The incorporation of antibacterial proteinoids into a natural biocompatible polymeric matrix can modulate its release, reducing the peak local concentration responsible for cytotoxicity. Moreover, the antibacterial material can be anchored to the surface of an inert support (such as silicate), reducing free diffusion in the biological matrix and limiting direct contact with eukaryotic cells, while maintaining local activity against bacteria. The design of a hybrid material can allow the antibacterial effect to be disconnected from the toxic effect through spatial and temporal control of release or through surface immobilization. In future studies, such strategies may be explored to optimize the balance between antimicrobial efficacy and biocompatibility. This study, therefore, not only provides first evidence of the antibacterial activity of proteinoids but also proposes a simple and economical synthetic approach to produce a new class of AMP-mimetic nanomaterials. Furthermore, it opens the way to the identification of promising individual antimicrobial peptides, which could be further optimized for specific applications.

## Figures and Tables

**Table 1:** Calculated percentages of pGlu in pure GluTyr products.

Sample	% *w*/*w* pGlu in Peptide Mixture
GluTyr 1:1	0.52 ± 0.07
GluTyr 2:1	0.22 ± 0.02
GluTyr 4:1	1.84 ± 0.2

## Data Availability

The original contributions presented in this study are included in the article/Supplementary Material. Further inquiries can be directed to the corresponding authors.

## Supplementary Materials

The following supporting information can be downloaded at https://www.mdpi.com/article/10.3390/nano15241846/s1. Figure S1. (a) FTIR absorption spectra of GluTyr products in the range of 3800–400 cm^−1^. Relevant amide vibrational modes are highlighted in the graph. (b) FTIR absorption spectra of tyrosine (Tyr), glutamic acid (Glu), pyroglutamic acid (pGlu), and GluTyr 1:1 in the range of 1900–400 cm^−1^. Dashed lines are a guide for relevant attributions; Figure S2. Molecular Weight Distribution of GluTyr 1:1 (magenta), 2:1 (green), and 4:1 (navy blue). Figure S3. ^1^H NMR spectra of (bottom): compound mixture from reaction between Glu and Tyr (1:1); (middle): compound mixture after D_2_O-shake; (top): pGlu. Figure S4. NMR COSY spectrum of compound mixture from reaction between Glu and Tyr (1:1). Figure S5. ^1^H NMR spectra of (bottom): compound mixture from reaction between Glu and Tyr (1:1); (top): compound mixture after D_2_O-shake. Figure S6. ^1^H NMR spectrum of compound mixture from reaction between Glu and Tyr (2:1). Figure S7. ^1^H NMR spectra of (bottom): compound mixture from reaction between Glu and Tyr (2:1); (top): compound mixture after D_2_O-shake. Figure S8. ^1^H NMR spectrum of compound mixture from reaction between Glu and Tyr (4:1). Figure S9. ^1^H NMR spectra of (bottom): compound mixture from reaction between Glu and Tyr (4:1); (top): compound mixture after D_2_O-shake. Figure S10. UV–visible absorption spectra of 3 mg mL^−1^ of pGlu, 0.05 mg mL^−1^ of L-Tyr, and 0.125 mg mL^−1^ of GluTyr products’ aqueous solutions. The concentrations were selected not to saturate the UV absorption, keeping the maximum absorption value under 3. At the same time, the concentration must not be too low in order to enlighten the absorption peaks. The inset graph shows details of the absorption spectra in the 300–450 nm range; Figure S11. The 3D-PL maps [excitation (y)–emission (x)–intensity (z)] in the 300–600 nm range of (a) pGlu 3 mg mL^−1^ and (b) Tyr 0.05 mg mL^−1^; Figure S12. LC-MS calibration curve for pyroglutamic acid. Note that all samples contained pGlu concentrations well within the linear portion of the graph. Figure S13. Minimum inhibitory concentration of pGlu against MRSA, determined by the broth microdilution method. Sterility control refers to the media without any bacterial inoculation. The experiment was performed with a biological replicate of 2, and in 3 technical replicates to ensure data integrity and reproducibility: Figure S14. Evaluated pH of the highest concentrations of GluTyr 1:1 and GluTyr 4:1 in DMEM during MTS cytotoxicity assay; Table S1. GluTyr 1:1 top 33 (of 48 elucidated) most abundant compounds differentiated by LC-MS using Agilent MassHunter Molecular Feature Extraction, with possible structures. Table S2. Comparison of retention times (RT) and abundances (% vol) of the top 20 most abundant compounds, and their possible structures, in each of the three Glu:Tyr reactant mixtures. The last 5 rows (grey italics) show compounds that were present in the top 20 of the 4:1 sample but not the top 20 of the others. Table S3. Calculated concentrations of pyroglutamic acid in peptide samples dissolved in 0.5 mL of water. Scheme S1. Published compounds with structures related to those proposed in the peptide mixture and with NMR analysis performed in DMSO-d6.
