# Supplementary material for: Antimicrobial Proteinoid Nanostructures via Thermal Condensation of L-Glutamic Acid and L-Tyrosine
Source: Nanomaterials (Basel). 2025 Dec 8;15(24):1846. doi: 10.3390/nano15241846 (PMC12736111; doi:10.3390/nano15241846)
Supplement: Supplementary file 1 [file nanomaterials-15-01846-s001.zip › nanomaterials-3976776-supplementary.pdf]

Supporting Information

Antimicrobial proteinoid nanostructures via thermal condensation of L-glutamic acid and L-tyrosine

Marta Cadeddu[1], James R. G. Adams[2], Roberto La Ragione[2,3], Daniel K. Whelligan[4], Vlad Stolojan[5], Nadia Bernardi[2], Ioannis Smyrnias[2], Barbara Poddesu[6], Giulia Cugia[6], Davide De Forni[6], Luca Malfatti[1], Davide Carboni[1]*, Alessandra Pinna[2,7]*, Plinio Innocenzi[1]*

[1] Laboratory of Materials Science and Nanotechnology, CR-INSTM, Department of Engineering, University of Sassari, Via Vienna 2, 07100 Sassari (SS), Italy.

[2] Department of Comparative Biomedical Sciences, School of Veterinary Medicine, Faculty of Health and Medical Sciences, University of Surrey, Guildford, UK.

[3] Discipline of Microbes, Infection and Immunity, School of Biosciences, Faculty of Health and Medical Sciences, University of Surrey, Guildford, UK.

[4] School of Chemistry and Chemical Engineering, University of Surrey, Guildford, UK.

[5] Advanced Technology Institute (ATI), School of Computer Science and Electronic Engineering, University of Surrey, Guildford, UK.

[6] ViroStatics S.r.l., Viale Umberto I, 46, 07100 Sassari (SS), Italy.

[7] Department of Materials, Imperial College London, London, United Kingdom.

Corresponding authors: dcarboni@uniss.it , plinio@uniss.it, a.pinna@surrey.ac.uk

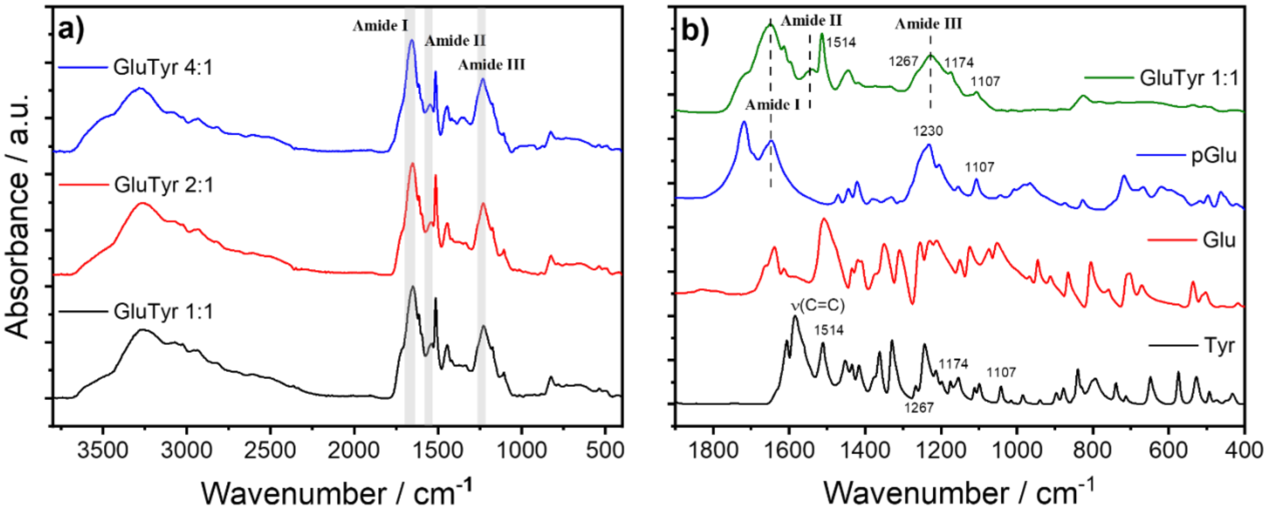

Figure S1. a) FTIR absorption spectra of GluTyr products in the range of 3800 – 400 cm^{-1}. Relevant amide vibrational modes are highlighted in the graph. **b)** FTIR absorption spectra of tyrosine (Tyr), glutamic acid (Glu), pyroglutamic acid (pGlu) and GluTyr 1:1 in the range of 1900 – 400 cm^{-1}. Dashed lines are a guide for relevant attributions

Table S1. GluTyr 1:1 top 33 (of 48 elucidated) most abundant compounds differentiated by LC-MS using Agilent MassHunter Molecular Feature Extraction, with possible structures.

Cmpd	Vol.	RT (min)	m/z	Mass	Mass calc.	Diff. (ppm)	Formula	Possible structure
**1**	23003214	6.135	454.1626	455.1693	455.1693	0.01	C23 H25 N3 O7	pGlu-Tyr-Tyr
**2**	19242624	4.495	291.0993	292.1059	292.1059	-0.17	C14 H16 N2 O5	pGlu-Tyr
**3**	17204448	4.897	291.0994	292.1061	292.1059	0.77	C14 H16 N2 O5	pGlu-Tyr isomer
**4**	9078511	5.912	454.1628	455.1701	455.1693	1.92	C23 H25 N3 O7	pGlu-Tyr-Tyr isomer
**5**	3363998	6.427	617.2259	618.233	618.2326	0.68	C32 H34 N4 O9	pGlu-Tyr-Tyr-Tyr
**6**	2481167	5.617	583.2047	584.2118	584.2118	0.01	C28 H32 N4 O10	pGlu-Tyr-Glu-Tyr
**7**	1977266	0.753	257.0785	258.0858	258.0852	2.3	C10 H14 N2 O6	pGlu-Glu
**8**	1598559	6.625	617.2256	618.2327	618.2326	0.2	C32 H34 N4 O9	pGlu-Tyr-Tyr-Tyr isomer
**9**	1399083	5.431	402.1311	403.1383	403.138	0.82	C19 H21 N3 O7	Glu-Tyr(pGlu)
**10**	1398316	4.007	291.0991	292.1063	292.1059	1.33	C14 H16 N2 O5	pGlu-Tyr isomer
**11**	1332602	6.132	628.2149	629.222				
**12**	1297408	1.736	420.1419	421.149	421.1485	1.16	C19 H23 N3 O8	pGlu-Glu-Tyr
**13**	1294077	6.532	325.1199	326.1271				
**14**	1067279	4.494	247.1091	248.1164				
**15**	1064944	2.923	291.0991	292.1063	292.1059	1.38	C14 H16 N2 O5	pGlu-Tyr isomer
**16**	997604	4.897	247.1092	248.1164				
**17**	966976	2.185	420.1414	421.1486	421.1485	0.2	C19 H23 N3 O8	pGlu-Glu-Tyr isomer
**18**	903831	1.304	420.1417	421.1489	421.1485	0.86	C19 H23 N3 O8	pGlu-Glu-Tyr isomer
**19**	783064	6.136	646.225	647.2322				
**20**	682112	4.5	465.1514	466.1586				
**21**	537787	3.064	420.1412	421.1484	421.1485	-0.27	C19 H23 N3 O8	pGlu-Glu-Tyr isomer
**22**	515266	4.909	465.1514	466.1587				
**23**	500894	5.603	454.1623	455.1695	455.1693	0.53	C23 H25 N3 O7	pGlu-Tyr-Tyr isomer
**24**	393653	4.495	605.1858	606.1931				
**25**	350930	6.799	780.2871	781.2943	781.2946	-0.4	C39 H41 N8 O10	pGlu-Tyr-Tyr-Tyr-Tyr
**26**	301806	4.899	605.186	606.1933				
**27**	297009	7.865	230.9862	231.9935				
**28**	275003	1.679	180.0666	181.0739				
**29**	267513	6.783	728.2556	729.2632	729.2646	-1.93	C33 H25 N22	pGlu-Tyr(pGlu)-Tyr-Tyr
**30**	228865	6.166	931.3113	932.3186				
**31**	208666	6.573	565.1935	566.2007	566.2013	-0.98	C28 H30 N4 O9	pGlu-Tyr(pGlu)-Tyr
**32**	181197	1.07	549.1832	550.1906	550.1911	-1.26	C24 H30 N4 O11	pGlu-Glu-Glu-Tyr
**33**	168830	0.696	386.1204	387.1277	387.1278	-0.67	C14 H15 N10 O4	pGlu-Glu-Glu

Table S2. Comparison of retention times (RT) and abundances (% vol) of the top 20 most abundant compounds, and their possible structures, in each of the three Glu:Tyr reactant mixtures. The last 5 rows (grey italics) show compounds that were present in the top 20 of the 4:1 sample but not the top 20 of the others.

Cmpd	Exact mass	Possible structure	GluTyr 1:1		GluTyr 2:1		GluTyr 4:1	
			RT	% vol	RT	% vol	RT	% vol
**1**	455.1693	pGlu-Tyr-Tyr	6.14	**25.2**	6.16	**28.3**	6.12	**18.5**
**2**	292.1059	pGlu-Tyr	4.50	**21.1**	4.59	**18.6**	4.29	**26.9**
**3**	292.1061	pGlu-Tyr isomer	4.90	**18.9**	4.99	**17.5**	4.77	**28.6**
**4**	455.1701	pGlu-Tyr-Tyr isomer	5.91	**10.0**	5.94	**9.6**	-	-
**5**	618.2330	pGlu-Tyr-Tyr-Tyr	6.43	**3.7**	6.45	**3.6**	-	-
**6**	584.2118	pGlu-Tyr-Glu-Tyr	5.62	**2.7**	5.67	**2.9**	5.59	**1.0**
**7**	258.0858	pGlu-Glu	0.75	**2.2**	0.75	**2.4**	-	-
**8**	618.2327	pGlu-Tyr-Tyr-Tyr isomer	6.63	**1.8**	6.64	**1.9**	6.61	**0.9**
**9**	403.1383	Glu-Tyr(pGlu)	5.43	**1.5**	5.48	**1.3**	5.40	**3.4**
**10**	292.1063	pGlu-Tyr isomer	4.01	**1.5**	4.02	**2.1**	3.86	**3.9**
**11**	629.2220		6.13	**1.5**	6.16	**1.7**	6.11	**1.1**
**12**	421.1490	pGlu-Glu-Tyr	1.74	**1.4**	1.73	**1.2**	1.73	-
**13**	326.1271		6.53	**1.4**	6.56	**1.4**	6.51	**2.5**
**14**	248.1164		4.49	**1.2**	4.59	**1.1**	4.29	**1.5**
**15**	292.1063	pGlu-Tyr isomer	2.92	**1.2**	2.91	**1.5**	2.89	**2.9**
**16**	248.1164		4.90	**1.1**	4.99	**1.0**	4.76	**1.6**
**17**	421.1486	pGlu-Glu-Tyr isomer	2.19	**1.1**	2.19	**1.1**	2.20	**0.9**
**18**	421.1489	pGlu-Glu-Tyr isomer	1.30	**1.0**	1.29	**1.1**	1.29	**1.1**
**19**	647.2322		6.14	**0.9**	6.16	**0.9**	6.11	-
**20**	466.1586		4.50	**0.7**	4.60	**0.8**	4.30	**0.9**
31	*566.2015*	*pGlu-Tyr(pGlu)-Tyr*					*6.55*	***1.7***
	403.1385						*6.04*	***2.4***
	351.1072						*2.63*	***1.6***
	274.0958						*6.17*	***1.3***
22	*466.1585*						*4.77*	***0.7***

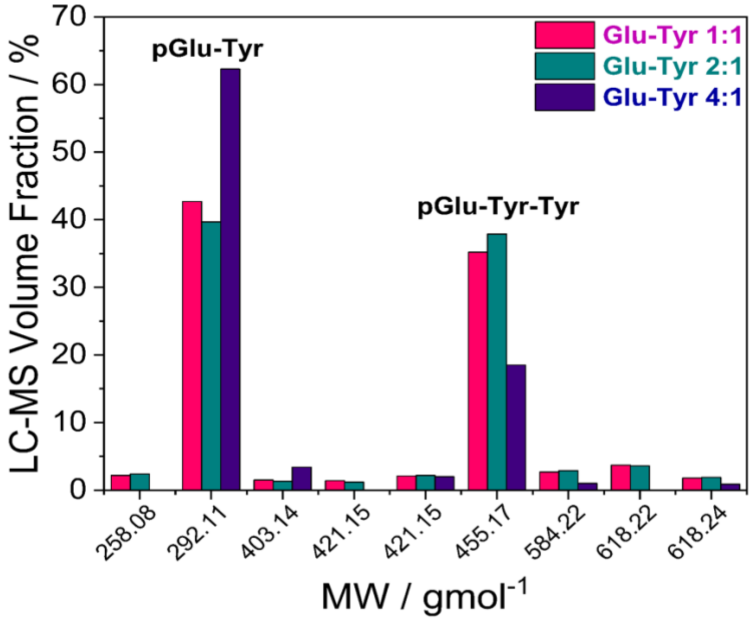

Figure S2. Molecular Weight Distribution of GluTyr 1:1 (magenta), 2:1 (green) and 4:1 (navy blue).

Explanation of NMR Assignments:

Compounds from the literature of related structure to those proposed to constitute our peptide mixtures are shown in **Scheme S1** and were chosen because ^{1}H NMR data, run in DMSO-d6, are reported by the authors.[1-3] By comparison to this data, and the NMR spectrum for pure pGlu (**Figure S3**) the peptide mixture NMR peaks around 2.0 ppm are most likely attributable to the CH_2 groups of pGlu and these are seen to correlate in a COSY spectrum (**Figure**) to the peak at 4.0 ppm which is therefore assigned to the pGlu CH. This also corresponds to the literature assignment of compound **C**. A D_2O shake (**Figure S**) saw the broad peak at 9.20 ppm almost disappear, indicating CO_2H, and the integral for the mixture of peaks around 8.0 ppm drop by 62% indicating exchangeable protons which are assigned to amide NHs. Again, this amide NH region corresponds with the literature assignment for compound **C**.

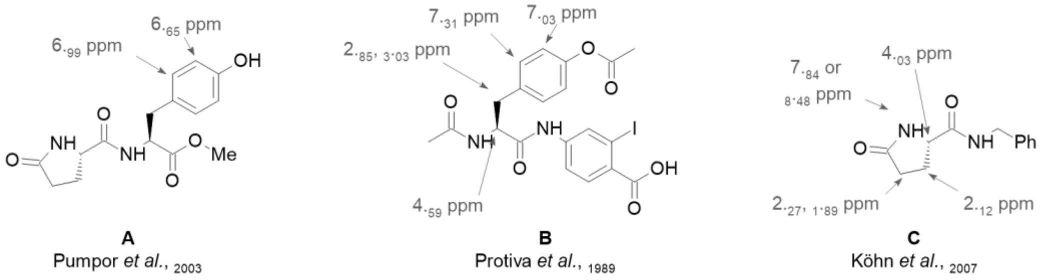

Scheme S1. Published compounds with structures related to those proposed in the peptide mixture and with NMR analysis performed in DMSO-d6.[1-3]

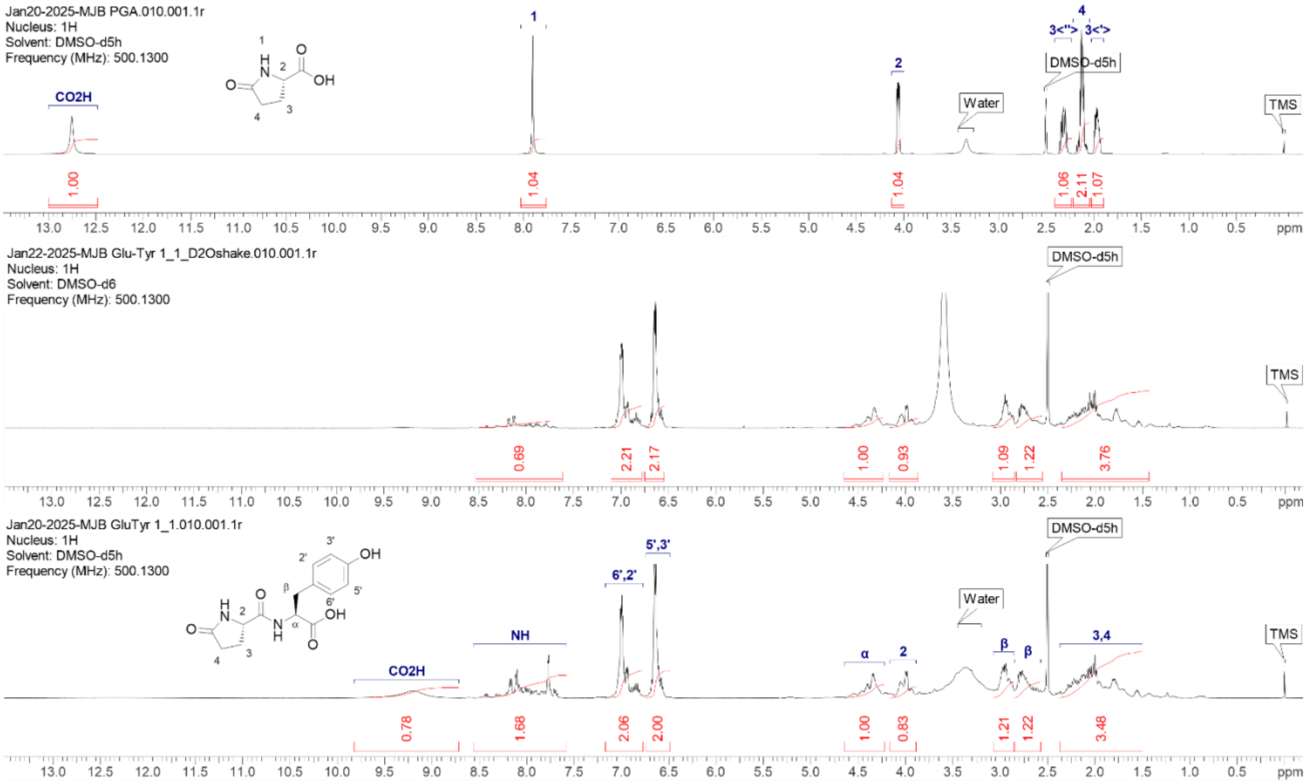

Figure S3. ^{1}H NMR spectra of (bottom): compound mixture from reaction between Glu and Tyr (1:1); (middle): compound mixture after D_2O-shake; (top): pGlu.

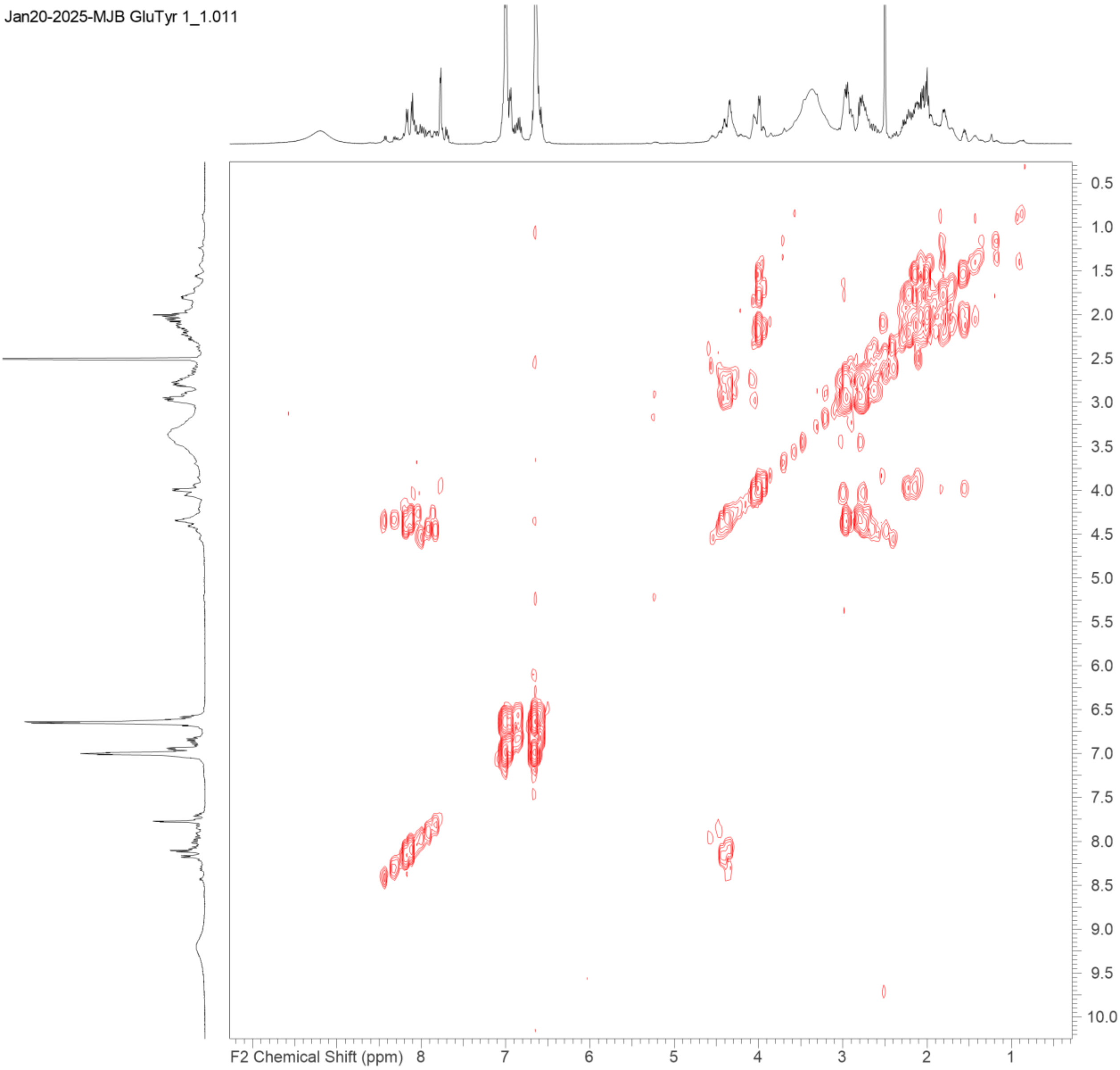

Figure S4. NMR COSY spectrum of compound mixture from reaction between Glu and Tyr (1:1).

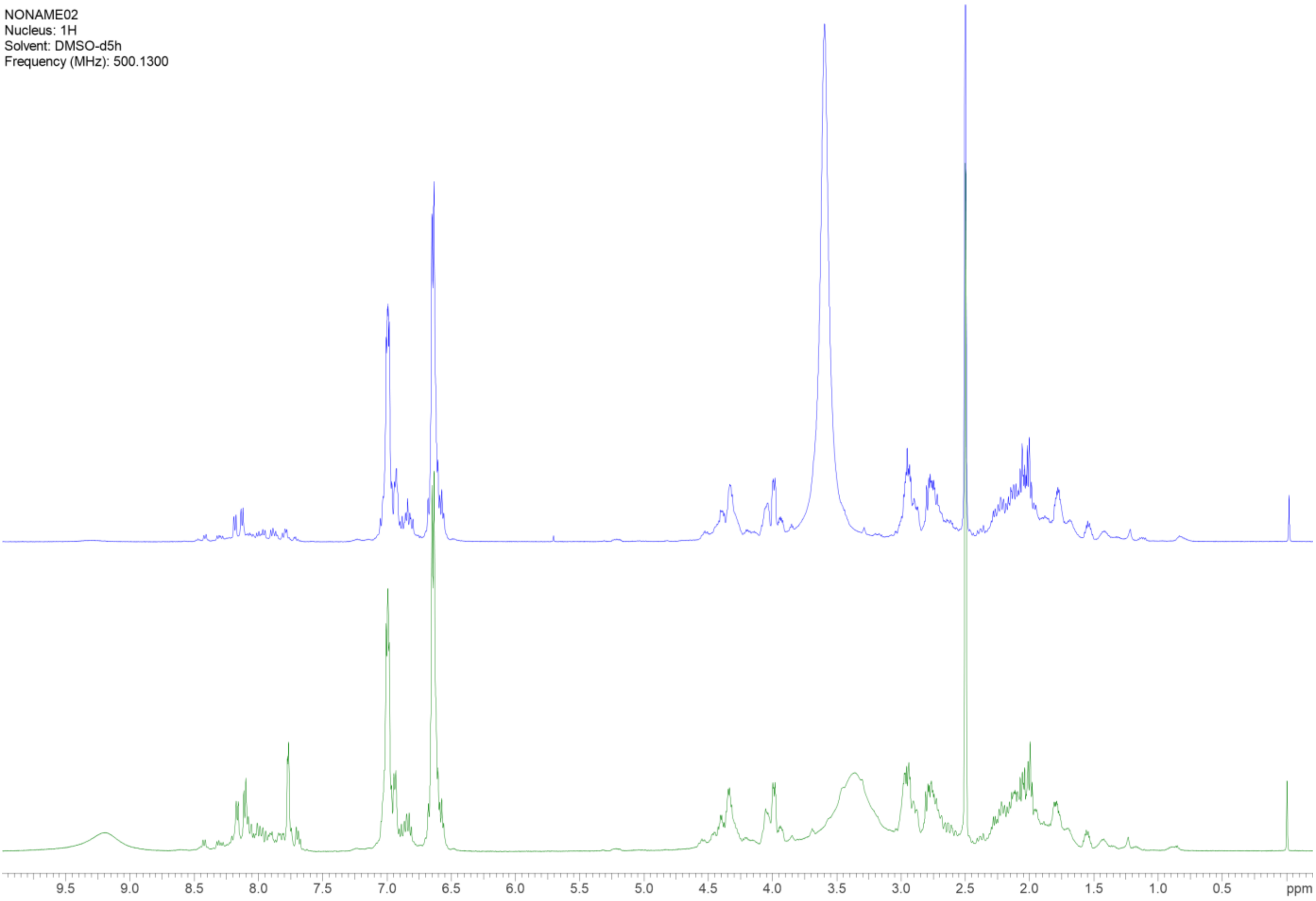

Figure S5. ^{1}H NMR spectra of (bottom): compound mixture from reaction between Glu and Tyr (1:1); (top): compound mixture after D_2O-shake.

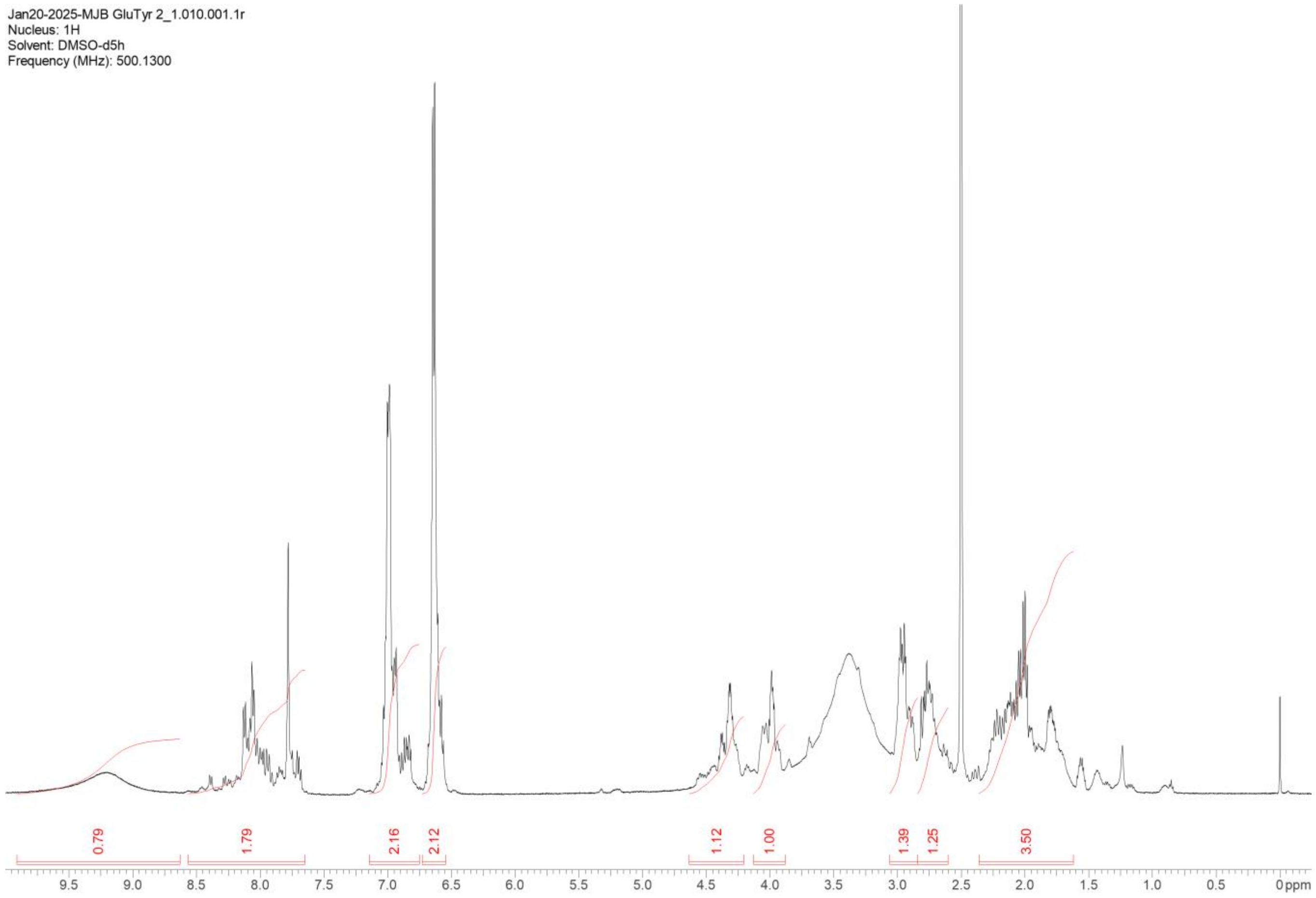

Figure S6. ^{1}H NMR spectrum of compound mixture from reaction between Glu and Tyr (2:1).

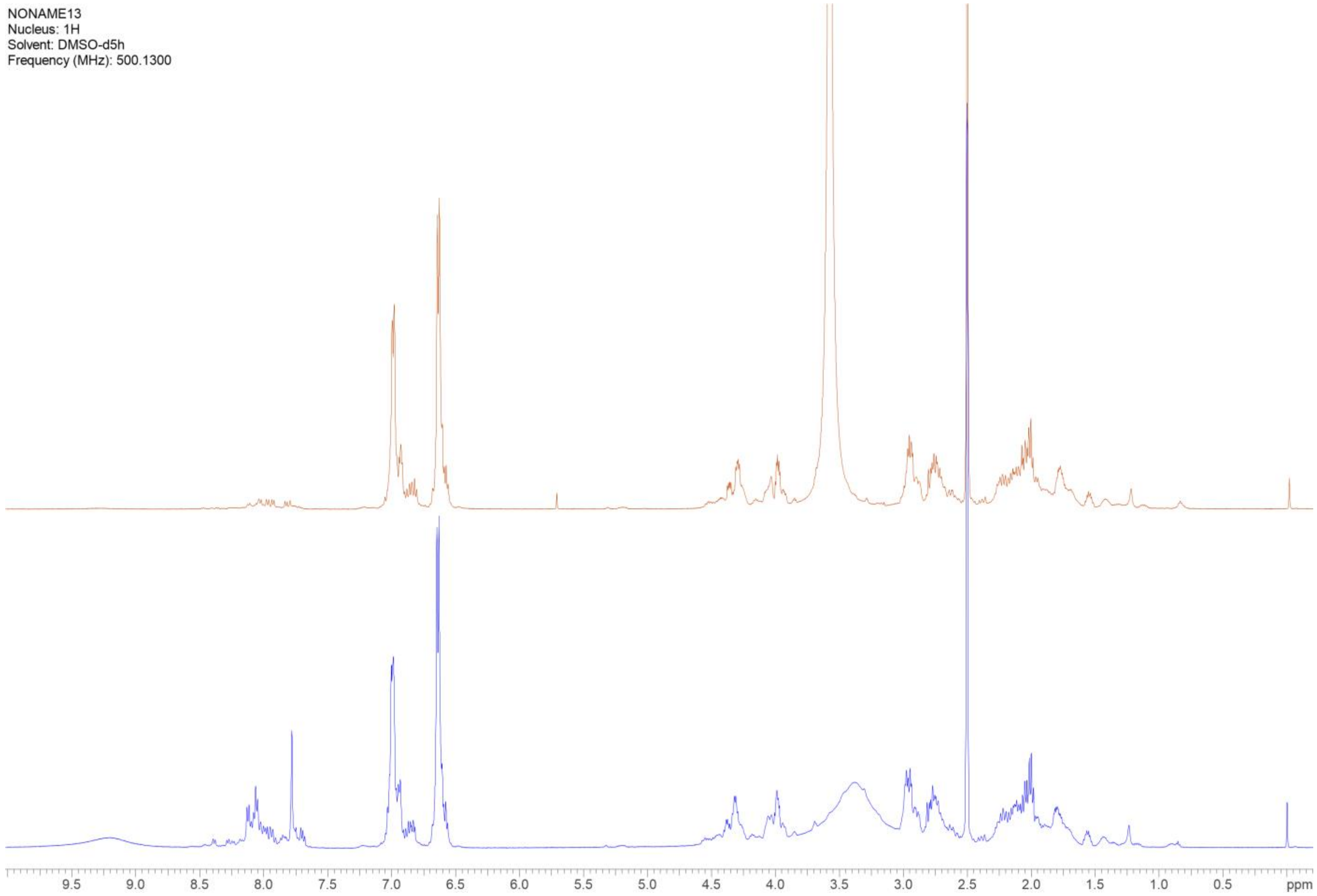

Figure S7. ^{1}H NMR spectra of (bottom): compound mixture from reaction between Glu and Tyr (2:1); (top): compound mixture after D_2O-shake

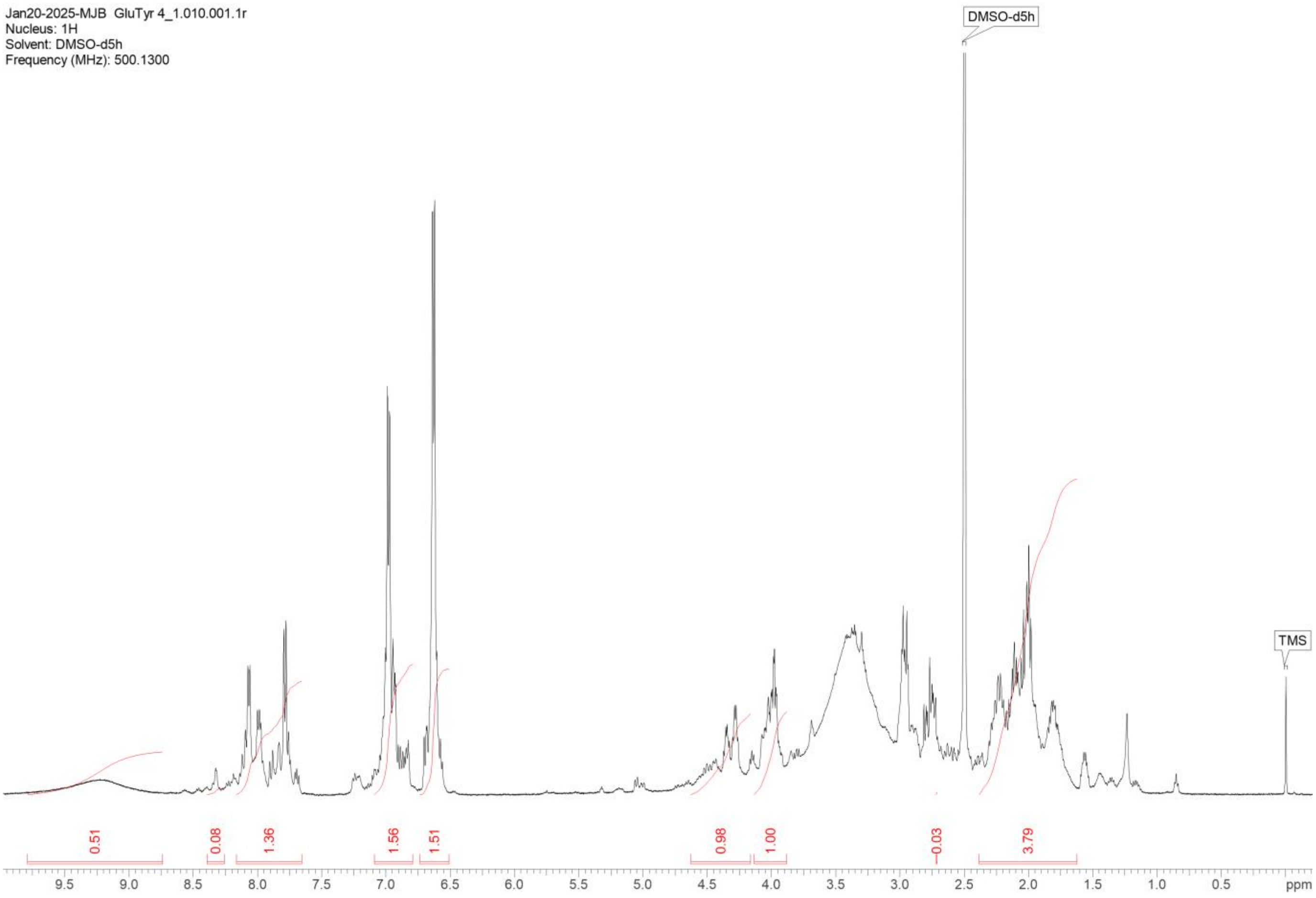

Figure S8. ^{1}H NMR spectrum of compound mixture from reaction between Glu and Tyr (4:1).

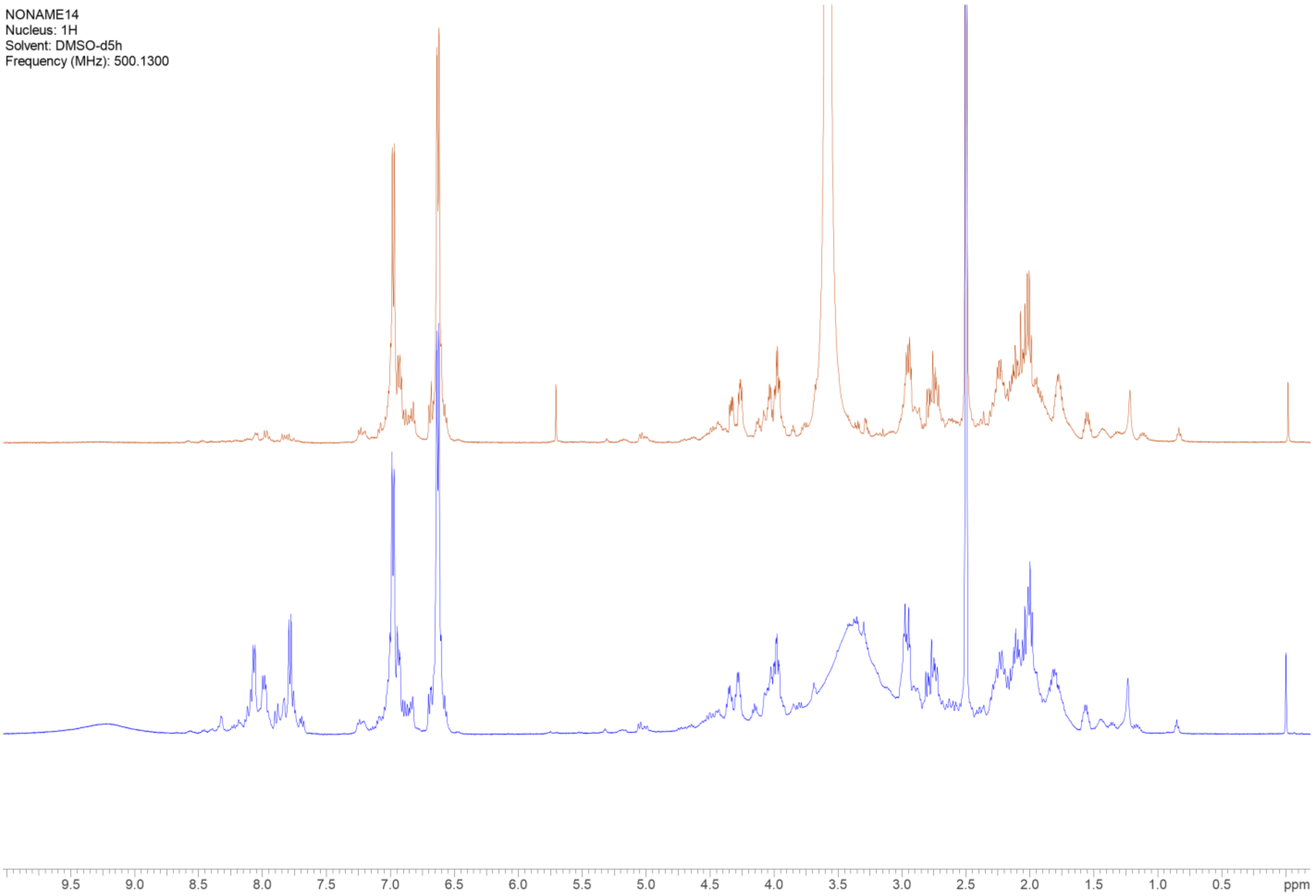

Figure S9. ^{1}H NMR spectra of (bottom): compound mixture from reaction between Glu and Tyr (4:1); (top): compound mixture after D_2O-shake.

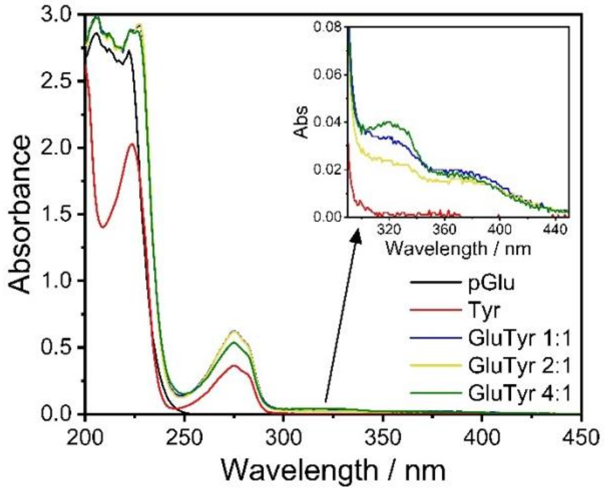

Figure S10. UV-Visible absorption spectra of 3 mg mL^{-1} of pGlu, 0,05 mg mL^{-1} of L-Tyr and 0,125 mg mL^{-1} of GluTyr products aqueous solutions. The concentrations are selected to not saturate the UV absorption, keeping the maximum absorption value under 3. At the same time, the concentration must not be too low in order to enlighten the absorption peaks. Inset graph shows details of the absorption spectra in the 300-450 nm range

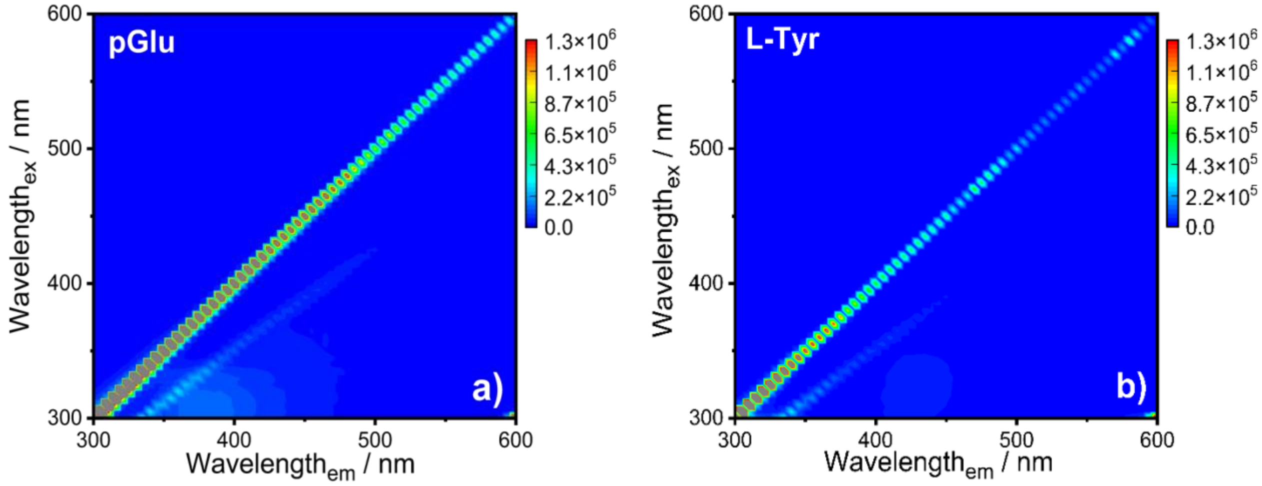

Figure S11. 3D-PL maps [excitation (y) − emission (x) − intensity (z)] in the 300-600 nm range of **a)** pGlu 3 mg mL^{-1} and **b)** Tyr 0.05 mg mL^{-1}.

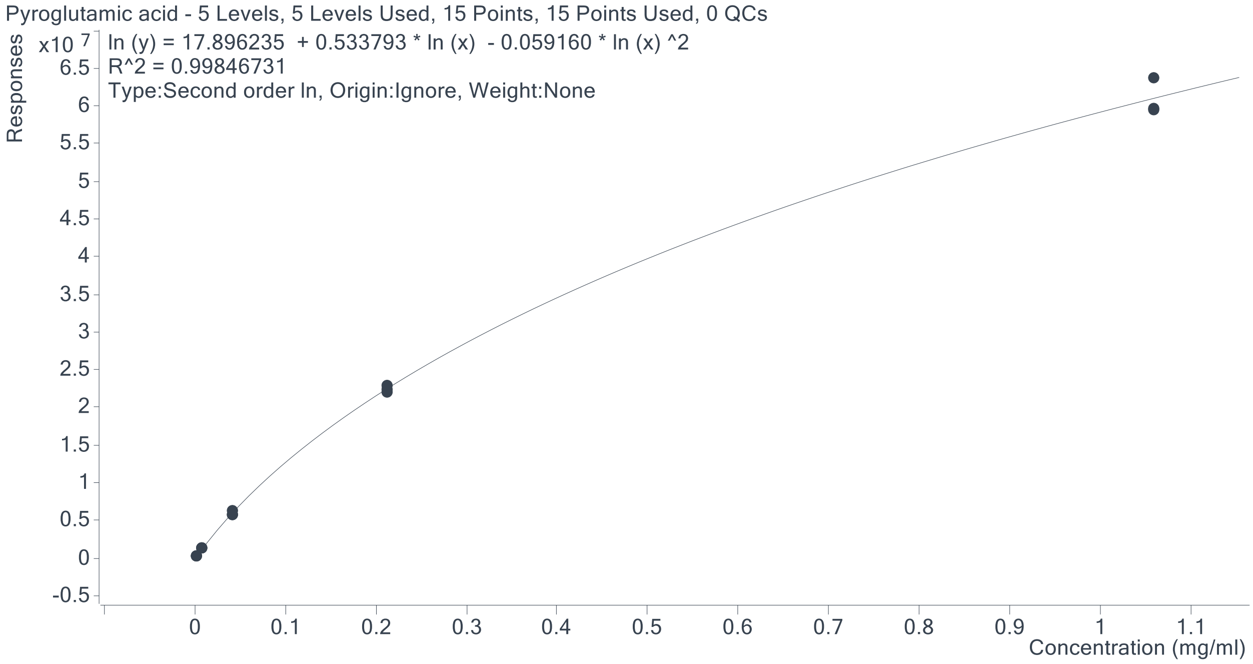

Figure S12. LC-MS calibration curve for pyroglutamic acid. Note all samples contained pGlu concentrations well within the linear portion of the graph.

Table S3. Calculated concentrations of pyroglutamic acid in peptide samples dissolved in 0.5 ml water.

Sample name	Mass peptide sample /mg	Conc rep 1 /mg/ml	Conc rep 2 /mg/ml	Conc rep 3 /mg/ml	Mean Conc /mg/ml	Conc SD /mg/ml	% w/w pGlu in peptide mixture
**GluTyr 1:1 0.586**	0.586	0.00543	0.00577	0.00694	0.00604	0.00079	*0.52 ± 0.07*
**GluTyr 2:1 0.54**	0.540	0.00215	0.00224	0.00261	0.00233	0.00025	*0.22 ± 0.02*
**GluTyr 4:1 0.559**	0.559	0.01854	0.02024	0.02306	0.02061	0.00228	*1.84 ± 0.2*

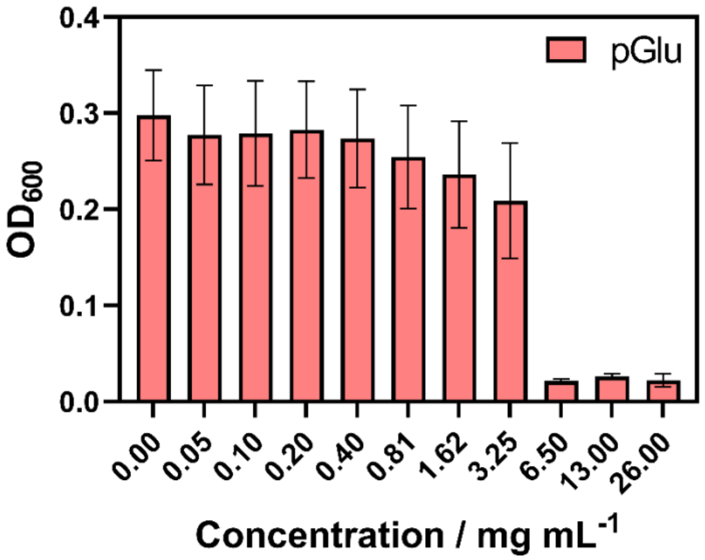

Figure S13. Minimum inhibitory concentration of pGlu against MRSA determined by the broth microdilution method. Sterile control, referring to the media without any bacterial inoculation, has been subtracted as background. The experiment was performed with a biological replicate of 2, and in 3 technical replicates to ensure data integrity and reproducibility.

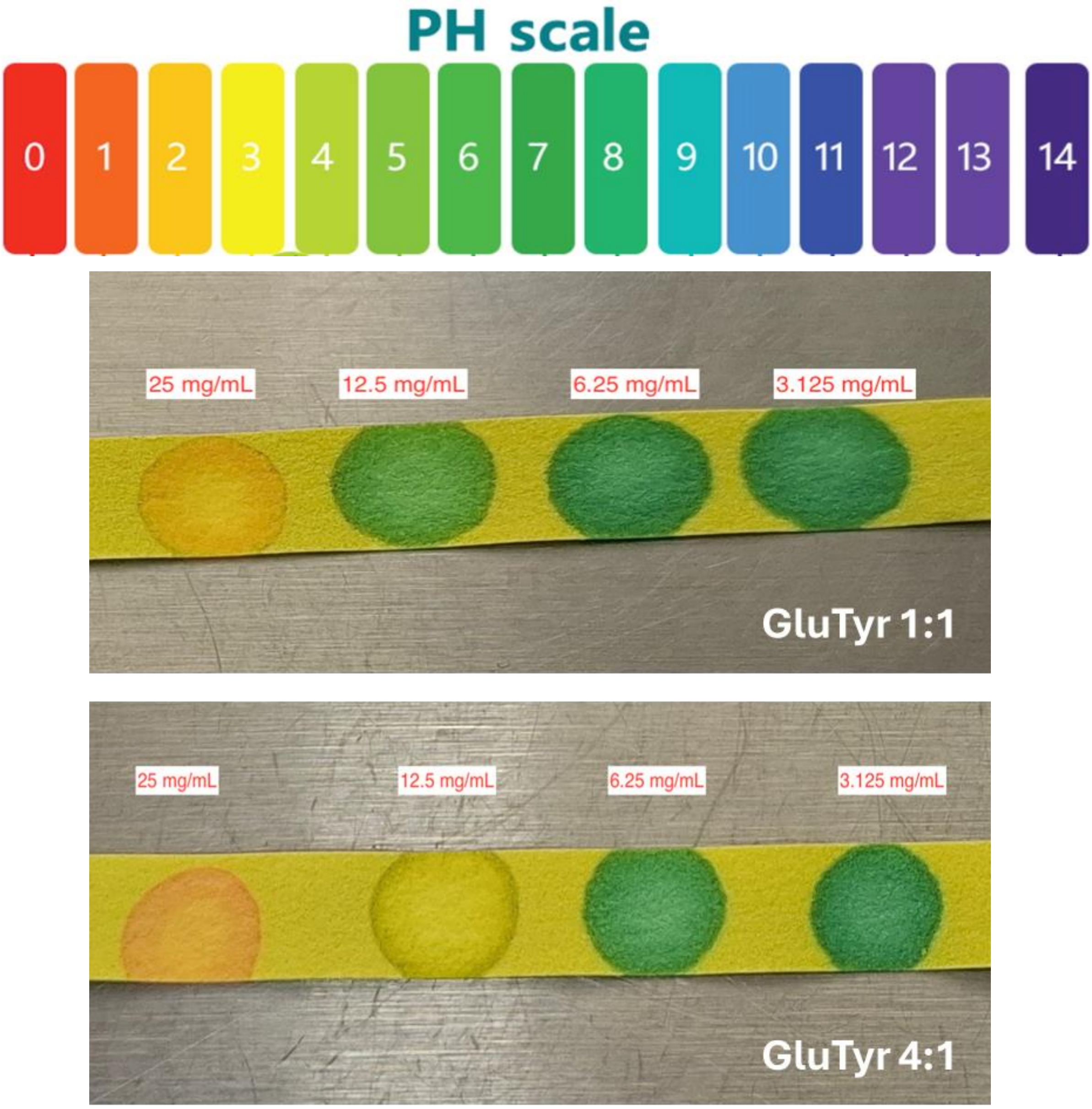

Figure S14. Evaluated pH of highest concentrations of GluTyr 1:1 and GluTyr 4:1 in DMEM during MTS cytotoxicity assay

References

1. Pumpor, K.; Böttcher, C.; Fehn, S.; Burger, K. Hexafluoroacetone as protecting and activating reagent: An efficient strategy for activation of pyroglutamic acid and homologues. *Heterocycles* **2003**, *61*, 259-269.
2. Protiva, J.; Krecek, V.; Maca, B.; Urban, J.; Budesinsky, M.; Prochazka, M. Synthesis of 4-(N-acetyl-L-tyrosyl)amino-2-iodobenzoic acid. *Collect. Czech. Chem. Commun.* **1989**, *54*, 1012-1018.
3. Köhn, U.; Schramm, A.; Kloß, F.; Görls, H.; Arnold, E.; Anders, E. Synthesis and characterization of chiral 1,2-diamines from 5-oxo-pyrrolidine-(S)-2-carboxylic acid. *Tetrahedron: Asymmetry* **2007**, *18*, 1735-1741.
